# Modulation of bone marrow haematopoietic stem cell activity as a therapeutic strategy after myocardial infarction: a preclinical study

**DOI:** 10.1038/s41556-025-01639-4

**Published:** 2025-04-02

**Authors:** Jasmin Rettkowski, Mari Carmen Romero-Mulero, Indranil Singh, Carolin Wadle, Jan Wrobel, Diana Chiang, Natalie Hoppe, Julian Mess, Katharina Schönberger, Maria-Eleni Lalioti, Karin Jäcklein, Beatriz SilvaRego, Timon Bühler, Noémie Karabacz, Mirijam Egg, Helen Demollin, Nadine Obier, Yu Wei Zhang, Claus Jülicher, Anne Hetkamp, Martin Czerny, Michael-Jason Jones, Hana Seung, Ritika Jain, Constantin von zur Mühlen, Alexander Maier, Achim Lother, Ingo Hilgendorf, Peter van Galen, Antonia Kreso, Dirk Westermann, Alejo E. Rodriguez-Fraticelli, Timo Heidt, Nina Cabezas-Wallscheid

**Affiliations:** 1 Max Planck Institute of Immunobiology and Epigenetics, Freiburg, Germany; 2 Spemann Graduate School of Biology and Medicine, Freiburg, Germany; 3 Faculty of Biology, University of Freiburg, Freiburg, Germany; 4 Laboratory of Stem Cell Biology and Ageing, Department of Health Sciences and Technology, ETH Zürich, Zurich, Switzerland; 5 Institute for Research in Biomedicine, Barcelona Institute for Science and Technology, Barcelona, Spain; 6 Facultat de Biologia, Universitat de Barcelona, Barcelona, Spain; 7 Department of Cardiology and Angiology, University Heart Center, Medical Center, University of Freiburg, Freiburg, Germany; 8 Faculty of Medicine, University of Freiburg, Freiburg, Germany; 9 Centre for Integrative Biological Signalling Studies, Freiburg, Germany; 10 International Max Planck Research School for Immunobiology, Epigenetics and Metabolism, Freiburg, Germany; 11 Department of Cardiovascular Surgery, University Heart Center, Medical Center, University of Freiburg, Freiburg, Germany; 12 Cardiovascular Research Center, Massachusetts General Hospital, Harvard Medical School, Boston, MA USA; 13 Institute of Experimental and Clinical Pharmacology and Toxicology, Faculty of Medicine, University of Freiburg, Freiburg, Germany; 14 Interdisciplinary Medical Intensive Care, Medical Center, Faculty of Medicine, University of Freiburg, Freiburg, Germany; 15 Division of Hematology, Brigham and Women’s Hospital and Harvard Medical School, Boston, MA USA; 16 Catalan Institution for Research and Advanced Studies, Barcelona, Spain

**Keywords:** Haematopoietic stem cells, Acute inflammation

## Abstract

Myocardial infarction (MI) is a major global health concern. Although myeloid cells are crucial for tissue repair in emergency haematopoiesis after MI, excessive myelopoiesis can exacerbate scarring and impair cardiac function. Bone marrow (BM) haematopoietic stem cells (HSCs) have the unique capability to replenish the haematopoietic system, but their role in emergency haematopoiesis after MI has not yet been established. Here we collected human sternal BM samples from over 150 cardiac surgery patients, selecting 49 with preserved cardiac function. We show that MI causes detrimental transcriptional and functional changes in human BM HSCs. Lineage tracing experiments suggest that HSCs are contributors of pro-inflammatory myeloid cells infiltrating cardiac tissue after MI. Therapeutically, enforcing HSC quiescence with the vitamin A metabolite 4-oxo-retinoic acid dampens inflammatory myelopoiesis, thereby modulating tissue remodelling and preserving long-term cardiac function after MI.

## Abstract (web summary)

Rettkowski, Romero-Mulero et al. show that myocardial infarction impacts bone marrow haematopoietic stem cells and leads to inflammatory myelopoiesis, which can be dampened by treatment with 4-oxo-retinoic acid, promoting cardiac recovery.

## Main

Myocardial infarction (MI) presents a substantial global health issue^1^. Post-MI survival and outcomes depend on acute compensatory responses, scar formation and tissue remodelling in both the cardiac lesion and remote myocardium. Inflammation, which is crucial for post-MI healing and tissue remodelling^2^, is driven by infiltrating leukocytes that coordinate processes such as debris breakdown, collagen deposition and neovascularization^3,4^. Demand for these inflammatory leukocytes after injury is met by emergency haematopoiesis (EH)^5–7^. However, excessive EH has been linked to worse remodelling, cardiac dysfunction and heart failure after MI^8,9^. Targeting systemic inflammation after MI has led to inconclusive results^10–14^. Still, there is an urgent need for new and specific therapeutic approaches.

Positioned at the apex of the haematopoietic system, bone marrow (BM) quiescent haematopoietic stem cells (HSCs) have the ability to generate multipotent progenitors (MPPs), which can differentiate into lineage-committed progenitors and subsequently give rise to leukocytes^15,16^. Dysregulation of HSC quiescence can lead to aberrant haematopoiesis such as clonal haematopoiesis and HSC exhaustion^17,18^. We have previously shown in mice that active metabolites of vitamin A are potent modulators of HSC activity, and they effectively safeguard HSCs against activation upon non-physiological stimuli^19,20^. It has been described that mouse HSCs proliferate and functionally decline upon MI^21–24^. Still, whether HSCs give rise to progeny during EH that infiltrate the heart upon MI has not been proven. In fact, the contribution of HSCs upon stress conditions has been recently challenged^25–27^.

In this preclinical study, we demonstrate that MI induces detrimental transcriptional and functional alterations in human BM HSCs. Furthermore, our study suggests that HSCs contribute to the generation of inflammatory cardiac-infiltrating leukocytes upon MI through lineage tracing experiments. We propose a therapeutic approach by modulating the root of EH with vitamin A metabolites, specifically forcing HSC quiescence in the aftermath of MI, to dampen the excessive EH and ultimately improve long-term cardiac function. We found all-*trans* retinoic acid (at-RA), a well-established clinical agent, to negatively impact local cardiac healing after MI, whereas its downstream metabolite, 4-oxo-retinoic acid (4-oxo-RA), bypassed these drawbacks and demonstrated improved cardiac recovery upon MI.

## Results

### MI leads to persistent activation and impaired functionality of human HSCs

**Fig. 1:**
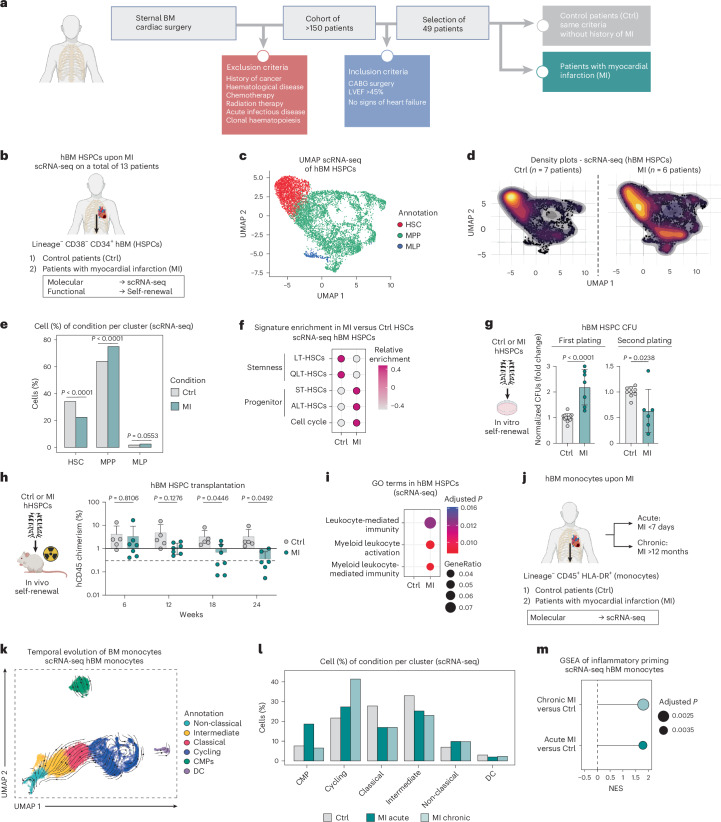
MI leads to persistent activation and impaired functionality of human HSCs. **a**, Patient selection flowchart. The diagram illustrates the process of patient cohort selection from initial BM collection to the final categorization into control patients (Ctrl) with no history of MI and those with MI. LVEF, left ventricular EF. **b**, Experimental design to characterize sternal human (h)BM HSPCs of MI and control donors. **c**, UMAP plot of scRNA-seq on human BM HSPCs upon MI coloured by cell type annotation. *n* = 7 Ctrl; *n* = 6 MI. **d**, UMAP density plots of control and MI-HSPCs depicting relative cell abundance. **e**, Bar plot of quantified relative cluster abundance in control and MI HSCs. Fisher tests. **f**, Relative enrichment scoring of published human HSPC signatures in control and MI HSCs. LT-HSCs, long-term HSCs; QLT-HSCs, quiescent long-term HSCs; ST-HSCs, short-term HSCs; ALT-HSCs, activated long-term HSCs. **g**, First and second plating of HSPC CFU assay comparing MI and control HSPCs. Two-tailed unpaired *t*-test. *n* = 9–11 Ctrl; *n* = 6–7 MI. **h**, Flow-cytometry-based analysis of human CD45 chimerism in HSPC transplantation assay of MI or control condition in NBSGW mice. The percentage of chimerism in PB was monitored over a time course of 24 weeks. Two-tailed unpaired *t*-test. *n* = 5 Ctrl; *n* = 7 MI. **i**, GO term enrichment of scRNA-seq MI and control DEGs (log_2_ fold change (FC) threshold 0.2, adjusted *P* value <0.1). **j**, Experimental design to characterize human sternal BM monocytes of MI acute, MI chronic and control donors. **k**, UMAP plot of scRNA-seq on human BM monocytes coloured by cell type annotation. The arrows show the RNA velocity vectors. *n* = 2 per condition. DC, dendritic cells. **l**, Bar plot of quantified relative cluster abundance in control, MI acute and MI chronic monocytes. **m**, GSEA of inflammatory-macrophage signature in MI acute and chronic versus control monocytes (merge of annotated clusters ‘classical’, ‘intermediate’ and ‘non-classical’). Data are presented as mean ± standard deviation. In **c**–**m**, *n* indicates the number of human BM donors (biological replicates). Source data

We collected sternal BM biopsies from >150 patients undergoing cardiac surgery (Fig. 1a). Patients with a history of cancer, haematological diseases, prior chemotherapy, clonal haematopoiesis or radiation therapy, infectious diseases and acute infections were excluded. We then selected patients with coronary artery bypass grafting (CABG) surgery and excluded cases with heart failure by means of reduced left ventricular ejection fraction (EF ≤45%) or other signs of cardiac congestion (for example, N-terminal pro-B-type natriuretic peptide (NT-proBNP) >1,000 pg ml^−1^). A total of 49 biopsies were used in the study, with patients falling into the category of either (1) control samples from patients with chronic coronary artery disease but no history of MI, or (2) samples from patients with a history of MI (Fig. 1a; detailed information available in Supplementary Table 1 and the Methods). To ensure comparability between the control and MI group, clinical patient characteristics such as age, gender and relevant health metrics were matched (Supplementary Table 1).

**Extended Data Fig. 1:**
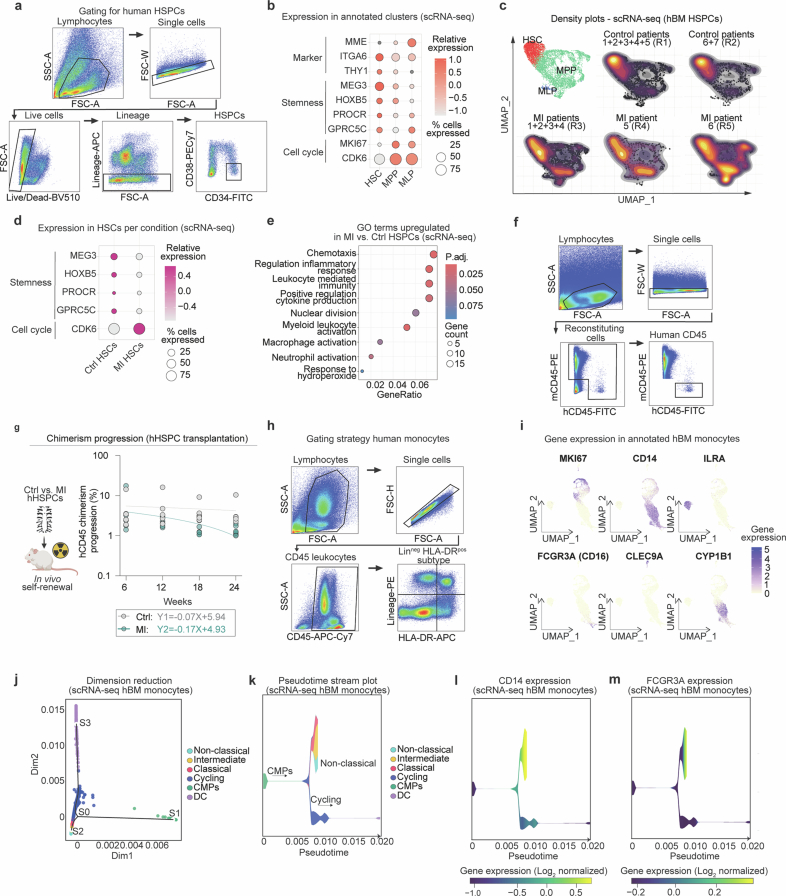
MI affects human bone marrow HSCs and monocytes. **a**, Representative gating scheme for flow cytometric cell sorting of human HSPCs. **b**, Relative expression dot plot of published human HSPCs markers in annotated clusters of human BM HSPCs scRNA-seq. Dot size corresponds to the percentage of cells expressing the depicted genes. **c**, UMAP scRNA-seq density plots of control and MI HSPCs in each experiment showing relative cell abundance. Pooled patients per reaction are depicted. R, Reaction. **d**, Relative expression dot plot of published human HSPCs markers comparing control and MI in the annotated HSC cluster of human BM HSCs scRNA-seq. Dot size corresponds to the percentage of cells expressing the depicted genes. **e**, GO terms enrichment of upregulated DEGs (log2FC threshold = 0.1, P.adj < 0.1) in human MI HSCs in comparison to control HSCs based on scRNA-seq. **e**, Representative gating scheme for flow cytometric analysis of human CD45 chimerism in PB in NBSGW mice. **f**, Representative gating scheme for flow cytometric analysis of human chimerism in transplanted NBSGW mice. **g**, Regression model of human CD45 chimerism in PB in NBSGW mice upon MI. **h**, Representative gating scheme for flow cytometric analysis of human monocytes in BM. **i**, UMAP plots showing expression levels of published human BM monocyte annotation markers in scRNA-seq. **j**, scRNA-seq on human BM monocytes ordered in low dimensional space together with trajectories inferred by STREAM. Cells are colored by the cell annotation. **k**, Stream plot showing cell density in different trajectories along pseudotime. Cells are colored by the cell annotation. **l**, Stream pseudotime plot colored by CD14 expression. **m**, Stream pseudotime plot colored by FCGR3A (CD16) expression. Source data

To determine the consequences of MI on human BM HSCs, we conducted single-cell RNA sequencing (scRNA-seq) on human stem and progenitor cells (HSPCs) (Lineage^neg^, CD38^neg^, CD34^pos^) isolated from seven patients with MI (MI-HSPCs) and six control patients (control-HSPCs) (Fig. 1b and Extended Data Fig. 1a). Annotation of scRNA-seq clusters revealed three major cell populations including HSCs, MPPs and multilymphoid primed progenitors (MLPs) (Fig. 1c, Extended Data Fig. 1b and Supplementary Table 2). Comparing the relative cell numbers between control and MI samples within each cluster, we found a significant decrease in HSCs, accompanied by increases in MPPs and MLPs, after MI (Fig. 1d,e and Extended Data Fig. 1c). Gene set enrichment scoring showed that stemness-associated gene signatures were downregulated, whereas terms related to cell-cycle activation were upregulated in HSCs upon MI (Fig. 1f, Extended Data Fig. 1d and Supplementary Table 2). Gene Ontology (GO) analysis highlighted upregulation of processes such as ‘regulation of inflammatory response’ and ‘positive regulation of cytokine production’, collectively suggesting increased cellular activity and differentiation in HSCs after MI (Extended Data Fig. 1e).

To assess the in vitro function of MI-HSPCs, we performed serial colony-forming unit (CFU) assays (Fig. 1g). MI-HSPCs showed increased colony output in the first plating, which was consistent with their higher level of activation and cell-cycle priming. Conversely, MI-HSPCs showed reduced colony formation in the second plating, indicating an impaired self-renewal capacity. To assess the in vivo functional potential, we transplanted human MI-HSPCs and their respective control into humanized mice NBSGW (NOD.Cg-KitW-41J Tyr+ Prkdcscid Il2rgtm1Wjl/ThomJ), an immunodeficient mouse model that allows engraftment of human HSCs (Fig. 1h). Human chimerism was monitored in peripheral blood (PB) for a period of 6 months, allowing the evaluation of long-term self-renewal capacity (Extended Data Fig. 1f). While both groups initially exhibited similar engraftment, MI-HSPCs demonstrated a reduced capacity to sustain long-term reconstitution (Fig. 1h and Extended Data Fig. 1g). These findings confirm an impaired functionality of human HSCs after MI.

GO term analysis showed that human MI-HSPCs were molecularly primed towards leukocyte activation, particularly towards myeloid cells (Fig. 1i). To further investigate the downstream consequences of HSC activation after MI, we focused on monocytes owing to their crucial role in cardiac injury^22^. We performed scRNA-seq analysis on BM monocytes (Lineage^neg^, CD45^pos^, HLA-DR^pos^) collected from patients with acute (MI <7 days at BM isolation) or chronic (MI >12 months at BM isolation) MI (Fig. 1j and Extended Data Fig. 1h). RNA velocity and pseudotime analysis suggested that annotated cells followed a trajectory from common myeloid progenitors (CMPs) to cycling cells, classical, intermediate and non-classical monocytes, successively (Fig. 1k, Extended Data Fig. 1i–m and Supplementary Table 3). During the acute phase, CMP levels significantly rose, suggesting an increased demand for monocyte production in response to cardiac injury, while the chronic phase revealed a rise in the subsequent cycling subpopulation (Fig. 1l). Notably, gene set enrichment analysis (GSEA) showed a pro-inflammatory priming during the acute phase that persisted into the chronic phase of post-MI classical monocytes (Fig. 1m and Supplementary Table 3). These findings suggest that MI leads to an inflammatory priming at the HSPC level, accompanied by chronic alterations in downstream monocyte progeny.

Collectively, our data provide compelling evidence of the activation and functional decline of human HSCs after MI.

### HSCs contribute to inflammatory myeloid cell infiltration in the heart

**Fig. 2:**
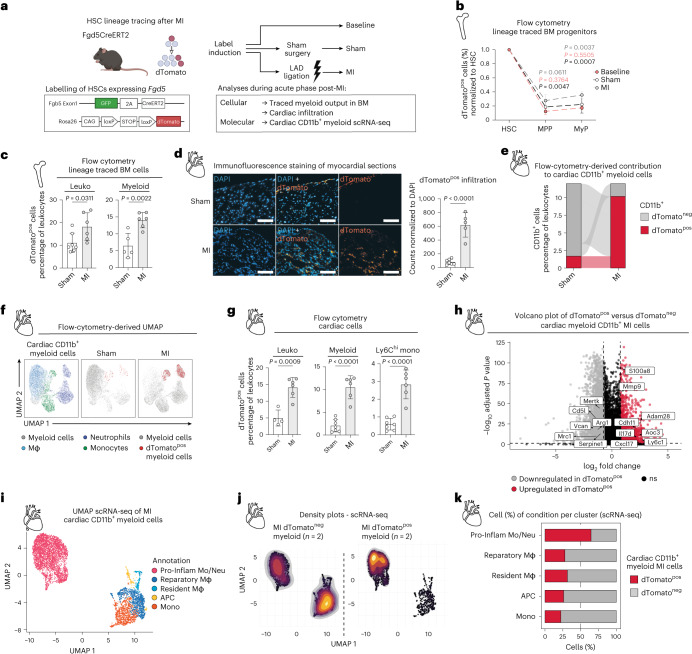
HSCs contribute to inflammatory myeloid cell infiltration in the heart. **a**, Experimental design for tracing the lineage of HSC responses following LAD artery ligation upon MI using the *Fgd5*^*CreERT2*^ mouse model. *Fgd5*^*CreERT2*^ mice express a tamoxifen-inducible CreERT2 recombinase and green fluorescent protein (ZsGreen) in the Fgd5 locus, which is highly active in HSCs. The expression of red fluorescent protein (tdTomato) is controlled by a loxP-flanked STOP cassette. Upon Cre-mediated recombination, dTomato fluorescence is observed. The dTomato expression is compared between (1) baseline mice undergoing induction without any surgical intervention, (2) non-ischaemic sham surgery mice and (3) LAD-ligated mice to simulate MI conditions. EH in *Fgd5*^*CreERT2*^ mice is evaluated during the acute phase at day 3 after MI. **b**, Flow-cytometry-based analysis of lineage-traced BM progenitors in *Fgd5*^*CreERT2*^ mice. The percentage of dTomato^pos^ cells is normalized to the dTomato label within the HSC compartment across the progenitors: HSCs, MPPs and MyP at baseline, after sham surgery and after MI. Ordinary two-way analysis of variance (ANOVA). Red *P* value, baseline versus sham; grey *P* value, sham versus MI; black *P* value, MI versus baseline. *n* = 6 vehicle; *n* = 6 sham; *n* = 3 baseline. **c**, Flow-cytometry-based plots, illustrating the percentage of lineage-traced dTomato^pos^ cell frequencies of myeloid cells upon MI or sham surgery in BM of *Fgd5*^*CreERT2*^ mice. Ordinary two-way ANOVA. *n* = 6 vehicle; *n* = 6 sham; *n* = 3 baseline. Leuko, leukocytes. **d**, Sections of myocardium stained with DAPI after MI. The counts of dTomato^pos^ cells are normalized to DAPI counts. Scale bar, 100 µm. Two-tailed unpaired *t*-test. *n* = 7 sham; *n* = 5 MI. **e**, Alluvial plot of contribution from dTomato^pos^ and dTomato^neg^, CD11b^pos^ cells to the myocardium upon MI. The *y* axis is reduced for visualization purposes. **f**, Flow-cytometry-based representative UMAP plots calculated on downsampled cells isolated from myocardium. The plots are representative for four sham mice and two MI mice. **g**, Flow-cytometry-based plots, illustrating the percentage of lineage-traced dTomato^pos^ leukocyte frequencies upon MI or sham surgery of *Fgd5*^*CreERT2*^ mice in the myocardium. Ordinary two-way ANOVA. *n* = 4 sham; *n* = 6 MI. Leuko, leukocytes; Ly6c2hi mono, Ly6c2-high monocytes. **h**, Volcano plot of DEGs between dTomato^pos^ and dTomato^neg^ mouse CD11b^pos^ cardiac MI cells in scRNA-seq. **i**, UMAP plot of scRNA-seq on mouse cardiac CD11b^pos^ cells in MI coloured by cell type annotation. Pro-Inflam Mo/Neu, pro-inflammatory monocytes and neutrophils; reparatory Mφ, reparatory macrophages; resident Mφ, resident macrophages; APC, antigen-presenting cells; Mono, monocytes. *n* = 2 per condition. **j**, UMAP density plots of dTomato^pos^ and dTomato^neg^ CD11b^pos^ cells in the myocardium upon MI depicting relative cell abundance. **k**, Stacked bar plot of quantified relative cluster abundance in dTomato^pos^ and dTomato^neg^ CD11b^pos^ cells in myocardium upon MI. Data are presented as mean ± standard deviation. In **b**–**i**, *n* indicates the number of biological replicates. For **b**–**e** and **g**, four independent experiments were performed. Source data

**Extended Data Fig. 2:**
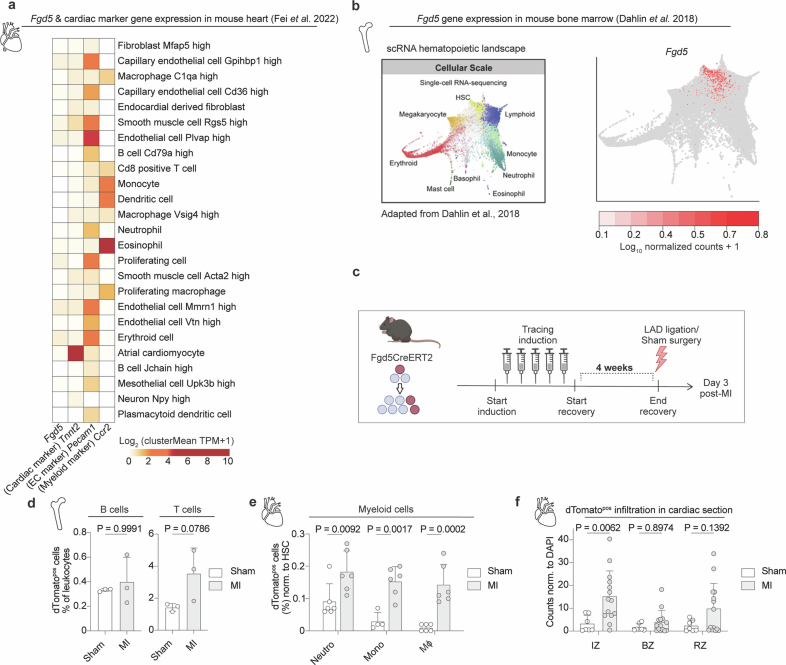
HSC-derived myeloid cells infiltrate the myocardium post-MI. **a**, Heatmap showing *Fgd5* and selected cardiac marker gene expression in all cell types from adult mouse heart, obtained from Fei et al., 2022. Expression data is presented as log2-transformed cluster mean transcripts per million (TPM + 1). **b**, t-SNE plot depicting *Fgd5* gene expression in scRNA-seq of mouse BM Lin^neg^ c-Kit^pos^ compartment from Dahlin et al., 2018. Expression levels are represented as log10(normalized counts + 1). **c**, Schematic design of the standard equilibrium tracing using Fgd5CreERT2 mice. Tracing is induced with tamoxifen injections, followed by a 4-week recovery before LAD ligation or sham surgery. Analysis is performed on day 3 post-MI. **d,e**, Flow cytometry-based plots, illustrating the percentage of lineage-traced dTomato^pos^ cells upon MI or sham surgery. **d**, Frequencies of dTomato^pos^ lymphoid cells in the BM. **e**, Frequencies of dTomato^pos^ myeloid cardiac cells are normalized to dTomato label within the corresponding HSC compartment of each mouse. Data is presented as mean with standard deviation. Neutro, Neutrophils; Mono, Monocytes; Macro, Macrophages. Ordinary two-way ANOVA. n = 6 for MI, sham condition. n = 3 for baseline condition. **f**, Stainings of cardiac sections, illustrating the infiltrating dTomato^pos^ cells upon MI or sham surgery in myocardium areas. Ordinary two-way ANOVA. n = 7-14. IZ, Infarct zone; BZ, Border zone; RZ, Remote zone. For (**d-f**) cells were isolated in the acute phase at day three after MI. Data are presented as mean ± standard deviation. n indicates the number of biological replicates per condition. For (**c**), three or more independent experiments were performed. Source data

Several studies have shown a correlation of mouse HSC proliferation and increased immune cells in the heart upon MI^22–24^. Previously, we observed that systemic anti-inflammatory treatment with IL-1β inhibitors was also associated with reduced HSC proliferation after MI^14^. However, it is still unknown whether these activated HSCs directly contribute to the immune progeny that infiltrates the heart tissue, and therefore it is unclear whether targeting HSC activation would be a reasonable therapeutic strategy for MI. To investigate whether HSCs are responsible for the production of infiltrating immune cells in the heart upon MI, we made use of the well-established *Fgd5*^*CreERT2*^ HSC fate mapping mouse model that has been used in numerous studies^25,28–31^. This mouse model harbours a ZsGreen-2A-CreERT2 cassette within the native *Fgd5* locus and is crossed with Rosa26-Lox-Stop-Lox-Tomato mice (*Rosa26*^*LSL-Tomato*^)^32^ (Fig. 2a). Tamoxifen administration results in a permanent dTomato label on HSCs that is inherited by their progeny, allowing lineage tracing. In the BM, *Fgd5* is highly expressed in HSCs compared with downstream haematopoietic populations^31^, while in the cardiac tissue we found only marginal expression in endothelial cells, validating its suitability for tracking HSC progeny in the context of MI (Extended Data Fig. 2a,b).

Upon label induction and after a standard equilibrium time of 4 weeks to minimize labelling variability in the HSC compartment, we subjected mice to MI by performing left anterior descending (LAD) artery ligation (Extended Data Fig. 2c). Sham surgery, which mimics the stress of the surgical procedure without inducing ischaemic injury, served as the control. Additional control groups included mice without any surgery or treatment, which served as a baseline for label equilibrium comparison.

After MI, we observed a significant increase in the proportion of dTomato-labelled (dTomato^pos^; HSC-derived cells) MPPs (Lineage^neg^, cKit^pos^, Sca-1^pos^ (LKS) CD48^pos^, CD150^neg^) and myeloid progenitors (MyPs; LKS^neg^) in the BM, as shown by flow cytometry (Fig. 2b). We used a mixed-effects linear model as previously reported, to evaluate the impact of MI on HSC differentiation across baseline, sham and MI conditions, with the baseline serving as the reference (Methods). To this end, data were normalized to the labelling in the HSC compartment. The sham condition showed no significant deviation from the baseline, suggesting that the observed variations in sham mice align with the expected range of the model, including random effects (indicated by a coefficient of 0.004; *P* = 0.865). By contrast, the MI condition showed a significant difference from the baseline, suggesting that MI drives a distinct differentiation response in cell compartments that cannot be solely attributed to the model and random effects (indicated by a coefficient of 0.083; *P* = 0.001). Subsequent analysis to determine which specific cell compartment contributes to this variation yielded non-significant results (*P* > 0.05) confirming that the observed changes in differentiation are attributable to a response from the source HSCs, rather than from other compartments (MPPs and MyP). These results suggest that MI increases the differentiation of HSCs through an MPP–MyP–myeloid trajectory. Furthermore, we observed increased absolute frequencies of dTomato^pos^ leukocytes (CD45^pos^), particularly myeloid cells (CD45^pos^, CD11b^pos^) upon MI, while lymphoid cells (CD45^pos^, B220^pos^ or CD3^pos^) did not change significantly (Fig. 2c and Extended Data Fig. 2d).

In the myocardium, we detected dTomato^pos^ cells by immunofluorescence staining (Fig. 2d), which particularly infiltrated the infarct zone after MI (Extended Data Fig. 2f). Furthermore, flow-cytometry-based quantification showed increased dTomato^pos^ leukocytes, especially myeloid cells including neutrophils (Lineage^neg^, CD45^pos^, CD11b^pos^, Ly6G^pos^), macrophages (Lineage^neg^, CD45^pos^, CD11b^pos^, Ly6G^neg^, F4/80^pos^) and inflammatory monocytes (Lineage^neg^, CD45^pos^, CD11b^pos^, Ly6G^neg^, F4/80^neg^, Ly6C^high^) (Fig. 2e–g and Extended Data Fig. 2e).

These findings indicate that BM HSC activation after MI triggers the production and subsequent infiltration of myeloid cells into the cardiac tissue.

**Extended Data Fig. 3:**
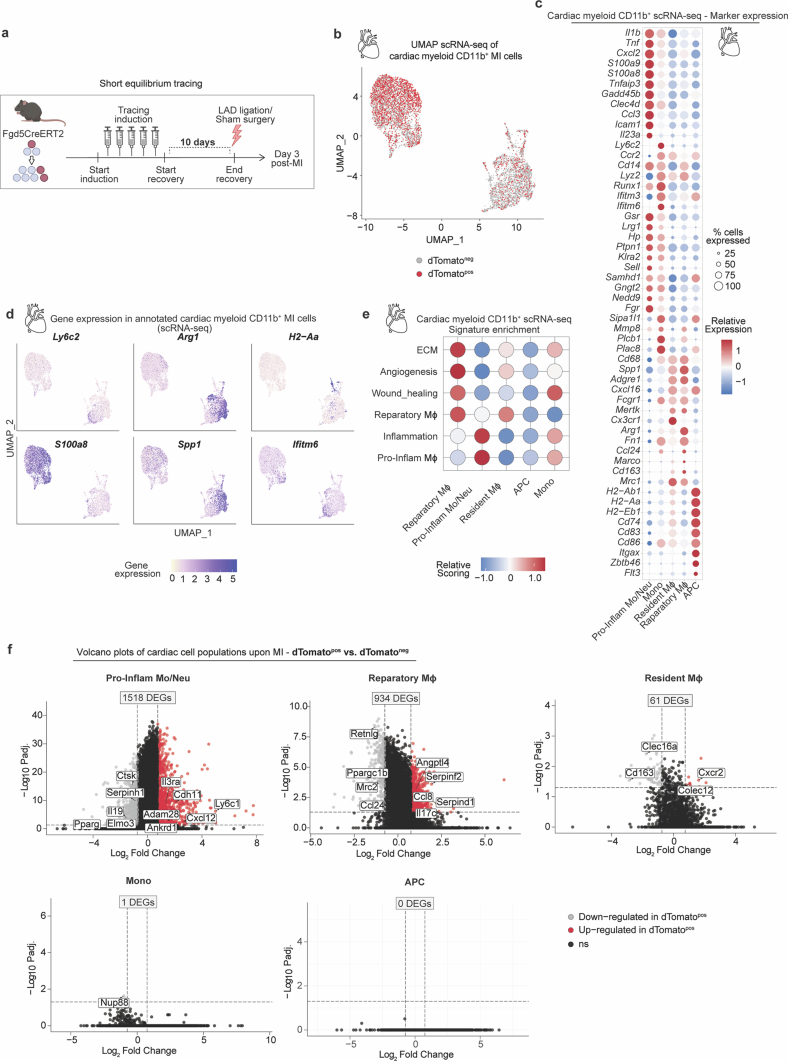
Post-MI HSC-derived cardiac myeloid cells show a pro-inflammatory transcriptional program. **a**, Schematic design of the short equilibrium tracing using Fgd5CreERT2 mice. Tracing is induced with tamoxifen injections, followed by a 10-day recovery before LAD ligation or sham surgery. Analysis is performed on day 3 post-MI. **b**, UMAP plot of scRNA-seq on mouse cardiac CD11b^pos^ cells in MI colored by dTomato phenotype. n = 2. **c**, Relative expression dot plot of published mouse cardiac myeloid annotation markers in annotated clusters of CD11b^pos^ (Fgd5CreERT2 mouse model) scRNA-seq. Dot size corresponds to the percentage of cells expressing the depicted genes. **d**, UMAP plots showing expression levels of published mouse cardiac CD11b^pos^ annotation markers in scRNA-seq. **e**, Relative scoring enrichment dot plot of published macrophage-related and inflammatory signatures in annotated clusters of mouse cardiac CD11b^pos^ scRNA-seq. **f**, Volcano plots of DEGs between dTomato^pos^ and dTomato^neg^ mouse CD11b^pos^ cardiac MI cells in each scRNA-seq subpopulation. Number of DEGs are depicted.

To minimize downstream labelling of HSC-derived cells at the time of MI, we shortened the equilibrium time (10 days) after labelling induction (Extended Data Fig. 3a). We then investigated HSC-derived cardiac myeloid infiltration by performing scRNA-seq on myeloid (CD11b^pos^) dTomato^pos^ and dTomato^neg^ cells, both isolated from the myocardium. We first assessed the overall gene expression changes between dTomato^pos^ and dTomato^neg^ myeloid cells. Interestingly, we observed an upregulation of inflammation and cardiac infiltration-related genes, such as *S100a8* and *Ly6c1*, in dTomato^pos^ myeloid cells, suggesting that they contribute to myocardial inflammation after MI (Fig. 2h and Supplementary Table 4). We then annotated the distinct myeloid subsets based on published population markers and gene signatures (Fig. 2i and Extended Data Fig. 3b–e). Quantification of cell abundance of each cluster showed that dTomato^pos^ cells are predominantly inflammatory monocytes and neutrophils (for example, high levels of *Tnf*, *Il1b* and *S100a8*; Fig. 2j,k)^22,33^, known for their central contribution to inflammation within the myocardium after MI. Notably, this cluster as well as reparatory macrophages showed the most transcriptomic differences, with a significant upregulation of pro-inflammatory genes such as *Ly6c1* and *Cxcl12* and downregulation of healing-related genes such as *Ccl24* and *Mrc2* in HSC-derived dTomato^pos^ infiltrating cells (Extended Data Fig. 3i and Supplementary Table 4).

Overall, we identified BM HSCs as contributors to the inflammatory myeloid cell infiltration in the myocardium after MI. These findings underscore the potential for targeted intervention at the HSC level to beneficially shape the subsequent immune response post-MI.

### Regulation of MI-HSCs by vitamin A metabolites

**Fig. 3:**
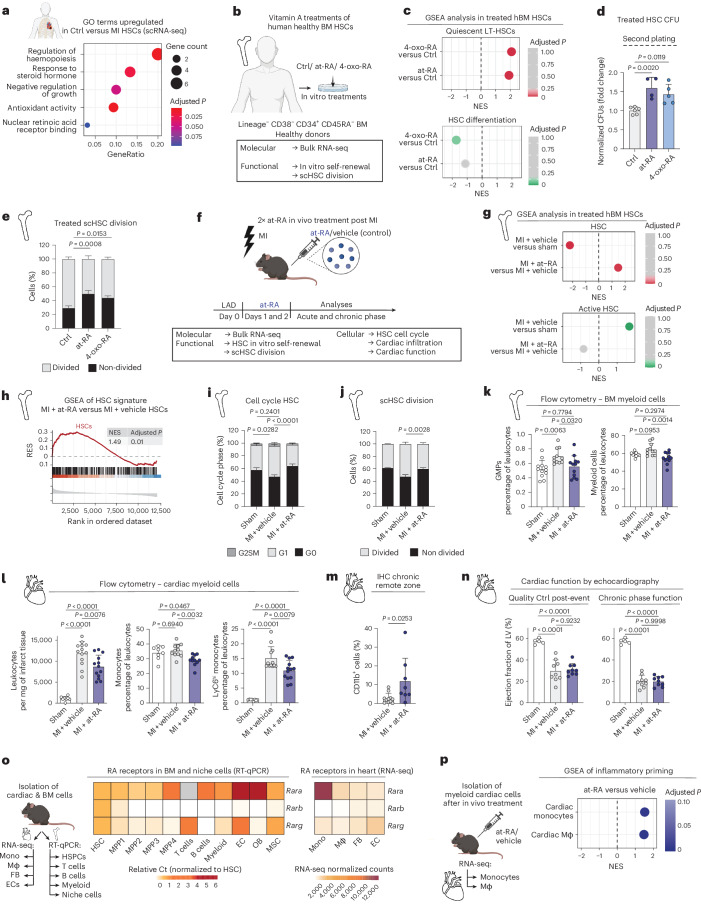
Regulation of MI-HCSs by vitamin A metabolites. **a**, GO term enrichment of upregulated DEGs (log_2_FC threshold 0.8, adjusted *P* value <0.05) in human control HSCs in comparison with MI HSCs based on scRNA-seq. **b**, Experimental design to characterize human BM HSCs isolated from healthy donors. HSCs were treated in vitro with RA metabolites (at-RA or 4-oxo-RA) or DMSO as control. **c**, GSEA of published human HSPC signatures in DEGs between 4-oxo-RA and at-RA treatments versus control from healthy human donors based on population RNA-seq. **d**, Second plating of CFU of human HSCs after in vitro cultivation with RA metabolites or control treatment. Ordinary one-way ANOVA. *n* = 6 Ctrl; *n* = 4 at-RA; *n* = 5 4-oxo-RA. **e**, scHSC division assay of HSCs isolated from BM healthy donors and in vitro cultured with RA metabolites or DMSO. Depicted *P* values correspond to the percentage of non-divided cells. Statistics denote comparisons between at-RA or 4-oxo-RA condition and the control condition. Ordinary two-way ANOVA. *n* = 7 Ctrl; *n* = 9 at-RA; *n* = 9 4-oxo-RA. **f**, Experimental design to characterize HSC response after at-RA in vivo treatment after MI. *n* = 4 vehicle; *n* = 3 at-RA; *n* = 2 sham. **g**, GSEA of published mouse HSPC signatures in vehicle versus sham HSCs in day-2 post-MI population RNA-seq. **h**, GSEA profile of HSC signature in at-RA versus vehicle HSCs upon MI. RES, running enrichment score. **i**, Flow-cytometry-based analysis of HSC cell cycle of sham, MI + vehicle and MI + at-RA conditions. The percentage of cell-cycle phases (G0, G1 and G2/S/M) is shown. Depicted *P* values correspond to the percentage of cells in the G0 phase. Statistics denote comparisons between vehicle or at-RA condition and the sham condition. Ordinary two-way ANOVA. *n* = 6 sham; *n* = 11 vehicle; *n* = 13 at-RA. **j**, scHSC division assay after 48 h in sham, MI + vehicle and MI + at-RA HSCs. The percentage of cells is shown. Depicted *P* values correspond to the percentage of non-divided cells. Statistics denote comparisons between vehicle or at-RA condition and the sham condition. Ordinary two-way ANOVA. *n* = 6 sham; *n* = 11 vehicle; *n* = 13 at-RA. **k**,**l**, Flow-cytometry-based analysis of leukocyte frequencies during EH in the acute phase at day 2 after MI. The percentage of leukocyte cell frequencies is depicted in BM and myocardium. Ordinary one-way ANOVA (**k**: *n* = 12 sham; *n* = 11 vehicle; *n* = 12 at-RA; **l**: *n* = 9 sham; *n* = 11 vehicle; *n* = 12 at-RA). GMPs, granulocyte-macrophage progenitors. **m**, Quantification of myocardium CD11b^pos^ immunohistochemistry (IHC) stainings of vehicle and at-RA condition in the chronic phase at day 28 after MI. Two-tailed unpaired *t*-test. *n* = 11 vehicle; *n* = 8 at-RA. **n**, The percentage of EF of LV based on echocardiography performed during the acute phase at day 1 after MI and during the chronic phase at day 21 after MI for each condition. Ordinary one-way ANOVA. *n* = 5 sham; *n* = 9 vehicle; *n* = 9 at-RA. LV, left ventricle. **o**, Expression profiles of RA receptors in BM and cardiac cells. Left: real time (RT)-qPCR in BM HSPCs, differentiated immune cells and niche cells. Normalized mean relative to *Oaz1* expression and relative to HSCs is shown. *n* = 3 per condition. Ct, cycle treshold. Right: heatmap of normalized counts in cardiac cell population RNA-seq dataset. *n* = 3 Mono; *n* = 4 Mφ; *n* = 3 FB; n = 4 EC. Mono, monocytes; Mφ, macrophages; FB, fibroblasts; EC, endothelial cells. **p**, GSEA of published mouse inflammatory-macrophage signature in cardiac monocytes and macrophages isolated from mice after 24 h in vivo treatment with at-RA or DMSO (vehicle), based on population RNA-seq. *n* = 3 Mono vehicle; *n* = 4 Mono at-RA; *n* = 4 Mφ vehicle; *n* = 3 Mφ at-RA. In **g**–**l**, cells were isolated in the acute phase at days 2–3 after MI. Data are presented as mean ± standard deviation. In **c**–**e**, *n* indicates the number of human BM donors per condition (biological replicates). In **g**–**p**, *n* indicates the number of biological replicates per condition. For **c**–**e** and **j**–**p**, two or more independent experiments were performed. Source data

To uncover potential regulators of MI-HSC activation, we performed GO term analysis of differentially expressed genes (DEGs) and identified a significant downregulation of retinoic acid (RA)/vitamin A receptor binding and hormone-related terms in human MI-HSCs (Fig. 3a and Supplementary Table 2). We have recently shown that RA metabolites such as at-RA and its downstream metabolite 4-oxo-RA positively modulate mouse HSC function by maintaining quiescence under stress conditions^19,20^. To explore the role of RA signalling as a modulator of human MI-HSCs, we first treated healthy human BM HSCs (Lineage^neg^, CD38^neg^, CD34^pos^, CD45RA^neg^) in vitro with at-RA or 4-oxo-RA and performed RNA-seq, CFU assays and single-cell division assays (Fig. 3b). In line with our mouse study, at-RA and 4-oxo-RA treatment increased transcriptional signatures associated with HSC features, enhanced in vitro self-renewal capacity and reduced the proportion of HSCs undergoing cell division (Fig. 3c–e). These findings show that RA metabolites positively regulate human HSC function. Given the observed dysregulation of RA signalling in human MI-HSCs (Fig. 3a), we hypothesized that these metabolites would be modulators of HSCs after MI.

**Extended Data Fig. 4:**
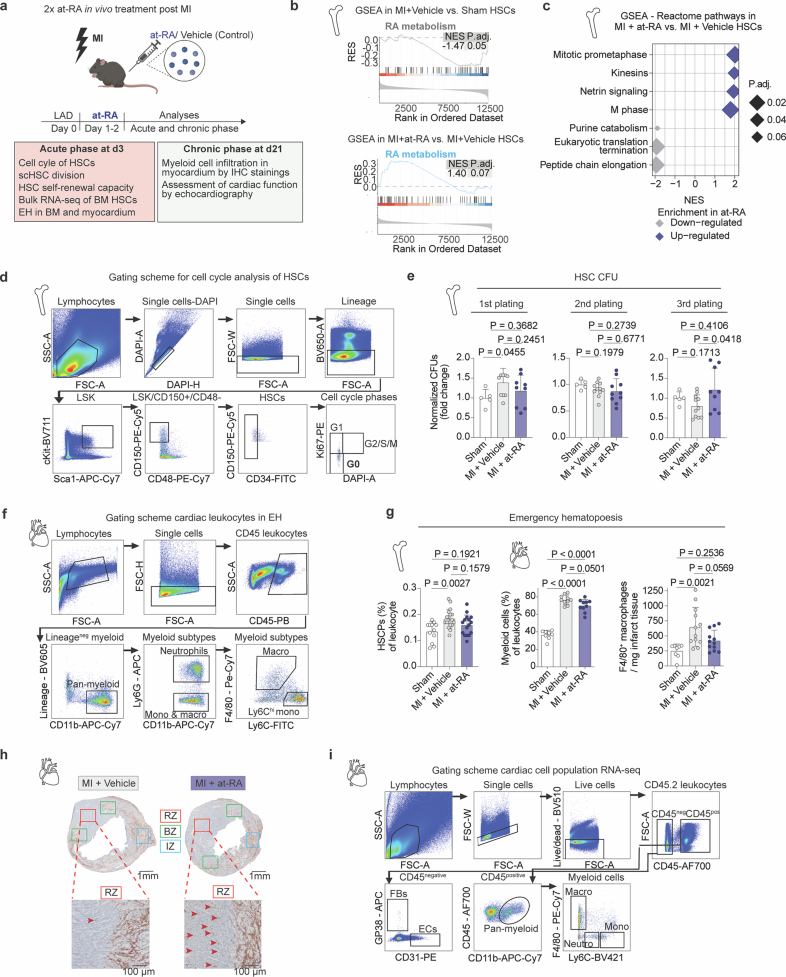
at-RA treatment modulates HSC activity post-MI but leads to accumulation of cardiac myeloid cells. **a**, Detailed experimental design to characterize HSC response after *in vivo* at-RA treatment following MI. Readouts are shown at precise time points throughout acute, reparative and chronic phases of MI. **b**, GSEA of mouse RA metabolism signature in pairwise comparisons vehicle vs. sham and at-RA vs. vehicle HSCs at day 2 post-MI population RNA-seq. **c**, GSEA of Reactome pathways in at-RA vs. vehicle HSCs upon MI. **d**, Representative gating scheme for flow cytometric cell cycle analysis of HSCs. **e**, 1^st^, 2^nd^ and 3^rd^ plating of HSC CFU assay comparing sham, MI+vehicle and MI+at-RA condition. Two-tailed unpaired t-test. n = 5–11. **f**, Representative gating scheme for flow cytometric analysis of cardiac leukocytes during EH. **g**, Left, Flow cytometry-based analysis of HSPC cell frequencies during emergency hematopoiesis in the acute phase at day 2 post MI in BM. Center, Flow cytometry-based analysis of myeloid cell and macrophage cell frequencies in the myocardium during emergency hematopoiesis in the acute phase at day 2 post MI in BM. Right, Cardiac macrophage frequency normalized to mg of infarct tissue is depicted. Ordinary one-way ANOVA. B(M) n = 8–18. **h**, Representative images of myocardium CD11b^pos^ immunohistochemistry (IHC) stainings of vehicle and at-RA condition in the chronic phase at day 28 post MI. RZ, Remote zone; BZ, Border zone; IZ, Infarct zone. Infiltrated CD11b^pos^ are pointed with an arrow. **i**, Representative gating scheme for flow cytometric isolation of cardiac cell populations isolated from mice after *in vivo* at-RA or vehicle (DMSO) treatment. In (**b-g**) cells were isolated in the acute phase at day 2-3 after MI. Data are presented as mean ± standard deviation. n indicates the number of biological replicates per condition. For (**e,g**), three or more independent experiments were performed. Source data

Due to the clinical availability of at-RA, we first investigated whether at-RA can modulate HSC activation in vivo upon MI and consequently reduce downstream immune cell production to ultimately enhance cardiac healing. We performed LAD ligation in mice followed by either at-RA or dimethyl sulfoxide (DMSO) (vehicle control) treatment for two consecutive days after MI (Fig. 3f and Extended Data Fig. 4a). Sham surgery served as a non-ischaemic control. We then analysed HSC activation in the acute phase after MI. GSEA revealed that HSCs were transcriptionally activated in vehicle-treated mice after MI compared with sham controls, whereas at-RA counteracted this activation and preserved stemness signatures in comparison with vehicle-treated mice (Fig. 3g,h and Extended Data Fig. 4b,c)^34^.

Cell-cycle analysis, ex vivo single-cell HSC (scHSC) division and CFU assays showed enhanced quiescence and in vitro self-renewal capacity of HSCs after MI upon at-RA treatment (Fig. 3i,j and Extended Data Fig. 4d,e). Overall, these findings demonstrate that at-RA effectively counteracts the functional decline of HSCs after MI.

We next assessed whether the beneficial effects of at-RA on HSCs extend to modulating the downstream immune response. We observed decreased leukocyte numbers in the BM, and myocardium in the acute phase of MI, including Ly6C^hi^ monocytes (Fig. 3k,l and Extended Data Fig. 4f,g). By contrast, immunohistochemistry staining of the myocardium revealed an accumulation of myeloid cells during the chronic phase after MI especially in remote areas distant to the initial lesion (Fig. 3m and Extended Data Fig. 4h). While echocardiographic analysis one day after surgery confirmed equal cardiac dysfunction between MI and at-RA-treated groups, cardiac function did not show any improvement upon at-RA treatment in the chronic phase after MI (Fig. 3n).

at-RA controls gene expression via the transcriptional activation of the three RA receptor (Rar) types, Rarα, Rarβ and Rarγ, exerting distinct transcriptional responses. To understand the mechanisms underlying the adverse effects of at-RA upon MI, we investigated the expression of RA receptors in the haematopoietic system (HSCs, MPPs, myeloid cells, T cells and B cells), the BM niche (endothelial cells, osteoblasts and mesenchymal stem cells) and the cardiac tissue (fibroblasts, endothelial cells, monocytes and macrophages). Real-time quantitative polymerase chain reaction (RT-qPCR) analysis showed that *Rara* and *Rarg* were highly expressed in several haematopoietic and BM niche populations, while *Rarb* was exclusively expressed in HSCs. RNA-seq of cardiac cell populations showed high *Rara* expression levels in monocytes and macrophages (Fig. 3o and Extended Data Fig. 4i). Considering Rarα’s established role in driving inflammation^35^ and the accumulation of cardiac myeloid cells upon at-RA treatment (Fig. 3m), activating the at-RA–Rarα axis in myeloid cells may counteract the beneficial effects of reducing HSC-induced EH on cardiac remodelling. In line with this, after treating wild-type mice with at-RA and performing RNA-seq on isolated cardiac monocytes and macrophages, we observed a global inflammatory priming in both populations (Fig. 3p).

Altogether, at-RA dampens HSC activation and reduces myelopoiesis upon MI while inducing a pro-inflammatory phenotype in cardiac myeloid cells, limiting the improvement in myocardial remodelling. Thus, the lack of specificity of at-RA precludes its usability to improve MI outcomes. Given that at-RA is a well-established clinical agent for haematological malignancies such as acute promyelocytic leukaemia and dermatological conditions, our findings suggest that the use of at-RA in patients with MI should be carefully considered.

### 4-oxo-RA safeguards HSC functionality upon MI

**Fig. 4:**
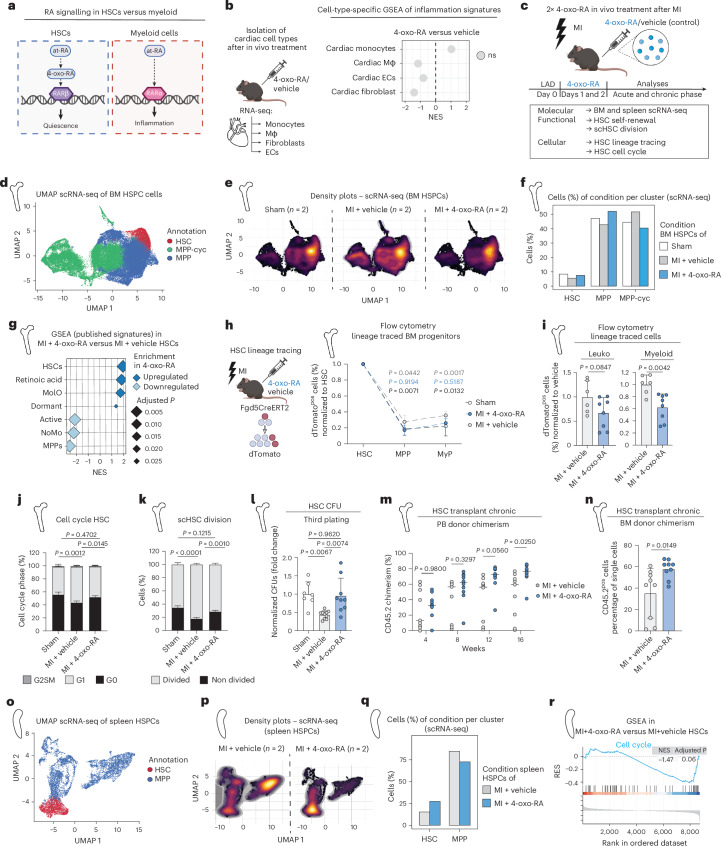
4-oxo-RA safeguards HSC functionality upon MI. **a**, Schematic representation of RA metabolite signalling (at-RA and 4-oxo-RA) comparing HSCs and myeloid cells. **b**, GSEA of published mouse inflammatory-macrophage signature in cardiac monocytes, macrophages (Mφ), endothelial cells (EC) and fibroblasts isolated from mice after 24 h in vivo treatment with 4-oxo-RA or DMSO (vehicle), based on population RNA-seq. *n* = 3 Mono vehicle; *n* = 3 Mono 4-oxo-RA; *n* = 4 Mφ vehicle; *n* = 4 Mφ 4-oxo-RA; *n* = 3 fibroblasts vehicle; *n* = 4 fibroblasts 4-oxo-RA; *n* = 4 ECs vehicle; *n* = 4 ECs 4-oxo-RA. ns, non-significant. **c**, Experimental design to characterize HSC response after 4-oxo-RA in vivo treatment following MI. **d**, UMAP plot of scRNA-seq on BM HSPC cells (Lineage^neg^, cKit^neg^, Sca1^pos^) isolated from mice that received in vivo treatment with vehicle (DMSO) or 4-oxo-RA after MI, as well as from mice undergoing sham surgery. *n* = 2 per condition. **e**, UMAP density plots of each condition in BM HSPC scRNA-seq depicting relative cell abundance. **f**, Quantification bar plot of relative cluster abundance in each BM HSPC scRNA-seq condition. Fisher tests. **g**, GSEA of published mouse HSPC signatures in 4-oxo-RA versus vehicle HSC cluster upon MI based on scRNA-seq. **h**, Flow-cytometry-based plots, illustrating the percentage of dTomato^pos^ cell frequencies of BM progenitors for sham, MI + vehicle and MI + 4-oxo-RA conditions in the BM. Each percentage is normalized to dTomato labelling within the corresponding HSC compartment. Data are presented as mean ± standard deviation. Ordinary two-way ANOVA. Black *P* value, sham vervsus MI + vehicle; blue *P* value, MI + vehicle versus MI + 4-oxo-RA; grey *P* value, MI + 4-oxo-RA versus sham. *n* = 5 sham; *n* = 6 vehicle; *n* = 8 4-oxo-RA. **i**, Flow-cytometry-based plots, illustrating the percentage of dTomato^pos^ cell frequencies for the vehicle and 4-oxo-RA conditions in the BM upon MI. Cell frequencies are normalized to the vehicle condition. Data are presented as mean ± standard deviation. Ordinary two-way ANOVA. *n* = 6 vehicle; *n* = 8 4-oxo-RA. **j**, Flow-cytometry-based analysis of HSC cell cycle of sham, MI + vehicle and MI + 4-oxo-RA conditions. The percentage of cell-cycle phases (G0, G1 and G2/S/M) is shown. Depicted *P* values correspond to the percentage of cells in the G0 phase. The statistics denote comparisons between vehicle or 4-oxo-RA condition and the sham condition. Ordinary two-way ANOVA. *n* = 7 sham; *n* = 10 vehicle; *n* = 11 4-oxo-RA. **k**, scHSC division assay after 48 h in sham, MI + vehicle and MI + 4-oxo-RA HSCs. The percentage of cells is shown. Depicted *P* values correspond to the percentage of non-divided cells. The statistics denote comparisons between vehicle or 4-oxo-RA condition and the sham condition. Ordinary two-way ANOVA. *n* = 7 sham; *n* = 10 vehicle; *n* = 11 4-oxo-RA. **l**, Third plating of HSC CFU assay comparing sham, vehicle and 4-oxo-RA conditions upon MI. Ordinary one-way ANOVA. *n* = 7 sham; *n* = 10 vehicle; *n* = 9 4-oxo-RA. **m**, Flow-cytometry-based analysis of CD45.2 PB chimerism in primary HSC transplantation assays of vehicle and 4-oxo-RA condition (CD45.2). The percentage of chimerism in PB was monitored over a time course of 16 weeks; radio-resistant cells were excluded. Ordinary two-way ANOVA. *n* = 9 vehicle; *n* = 10 4-oxo-RA. **n**, Flow-cytometric analysis of CD45.2 chimerism at 16 weeks in BM. Two-tailed unpaired *t*-test. *n* = 9 per condition. **o**, UMAP plot of scRNA-seq on mouse spleen HSPC cells isolated from vehicle and 4-oxo-RA conditions upon MI. *n* = 2 per condition. **p**, UMAP density plots of vehicle and 4-oxo-RA spleen HSPC scRNA-seq upon MI depicting relative cell abundance. **q**, Bar plot of quantified relative cluster abundance in vehicle and 4-oxo-RA spleen HSPC scRNA-seq upon MI. **r**, GSEA profile of cell-cycle pathway in 4-oxo-RA versus vehicle HSCs scRNA-seq upon MI. In **d**–**l** and **o**–**r**, cells were isolated in the acute phase at day 3 after MI. Data are presented as mean ± standard deviation. *n* indicates the number of biological replicates per condition. For **b** and **h**–**n**, three or more independent experiments were performed. Source data

**Extended Data Fig. 5:**
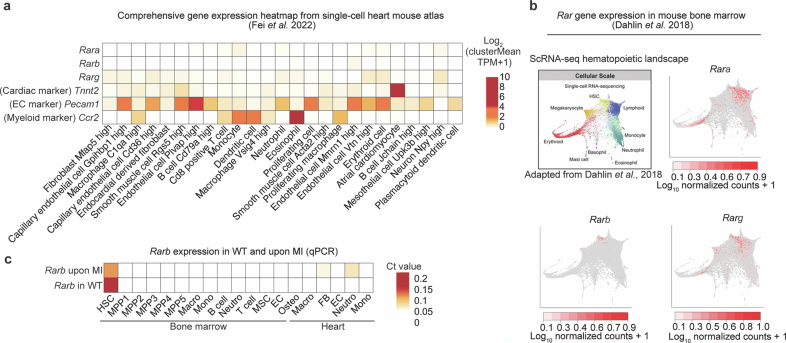
*Rarb* expression is specific to HSCs and remains unchanged upon MI. **a**, Heatmap illustrating the expression of RA receptor genes and cardiac, endothelial, and myeloid marker genes in all cell types from adult mouse heart, obtained from Fei et al., 2022. Expression levels are indicated by the log2-transformed cluster mean TPM + 1. **b**, t-SNE plot illustrating *Rara*, *Rarb* and *Rarg* gene expression in scRNA-seq of mouse BM Lin^neg^ c-Kit^pos^ compartment from Dahlin et al., 2018. Expression levels are represented as log10(normalized counts + 1). **c**, qPCR expression profile of *Rarb* in bone marrow and cardiac populations in control and MI mice. Normalized mean relative to *Oaz1* housekeeper expression is shown. n = 3-4. In **c**, cells were isolated 24 h after MI.

**Extended Data Fig. 6:**
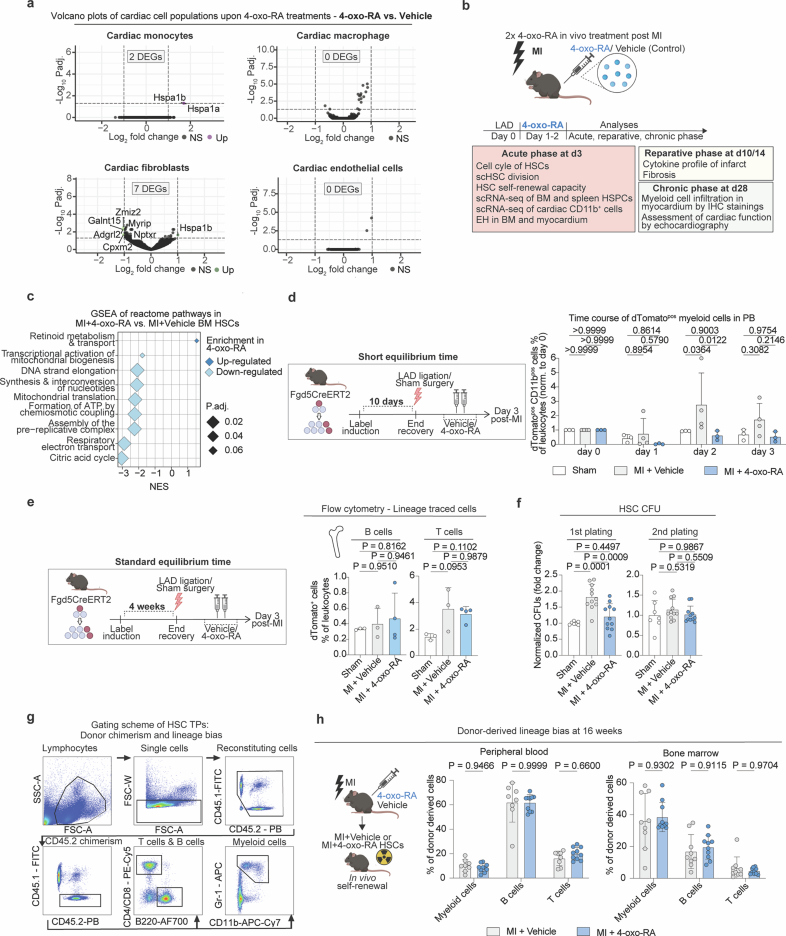
4-oxo-RA safeguards HSCs upon MI. **a**, Volcano plots of DEGs between 4-oxo-RA and DMSO (vehicle) treatment depicted for cardiac monocytes, macrophages, fibroblasts and endothelial cells. **b**, Detailed experimental design to characterize HSC response after *in vivo* 4-oxo-RA treatment following MI. Readouts are shown at precise time points throughout acute, reparative and chronic phases of MI. **c**, GSEA of Reactome pathways in 4-oxo-RA vs. vehicle BM HSC cluster upon MI based on scRNA-seq. **d**, Flow cytometry-based plots, illustrating the percentage of BM lineage-traced dTomato^pos^ lymphoid cells upon MI or sham surgery and treatment in our short equilibrium tracing. Data is presented as mean with standard deviation in different timepoints. Ordinary two-way ANOVA. n = 3–4. **e**, Flow cytometry-based plots, illustrating the percentage of BM lineage-traced dTomato^pos^ lymphoid cells upon MI or sham surgery and treatment in our standard equilibrium tracing. Data is presented as mean with standard deviation. Ordinary two-way ANOVA. n = 3–4. **f**, 1^st^ and 2^nd^ plating of HSC CFU assay comparing sham, vehicle and 4-oxo-RA conditions upon MI. Ordinary one-way ANOVA. n = 7–11. **g**, Representative gating scheme for flow cytometric analysis of HSC transplantation assay of 4-oxo-RA and vehicle condition. Percentage of CD45.2 PB chimerism was monitored over a time course of 16 weeks. Percentage of donor-derived lineage bias in B-cells, myeloid cells and T-cells was quantified at 16 weeks in BM. **h**, Flow cytometric analysis of donor-derived (CD45.2) lineage bias in B-cells, myeloid cells and T-cells in PB and BM at 16 weeks post transplantation. Ordinary two-way ANOVA. n = 8–10. In (**c, e**), cells were isolated in the acute phase at day 3 after MI. Data are presented as mean ± standard deviation. n indicates the number of biological replicates per condition. For (**a, e** and **f**), two or more independent experiments were performed. Source data

We previously showed that 4-oxo-RA, a downstream metabolite of at-RA, promotes HSC quiescence through Rarβ binding^20^. *Rarb* is not expressed in cardiac cells, while in the BM *Rarb* is exclusively expressed in HSCs compared with downstream progenitors, all differentiated blood cells including monocytes, and BM niche cells, as assessed by both transcriptomics and RT-qPCR (Fig. 3o and Extended Data Fig. 5a–c). Moreover, its expression is not affected upon MI (Extended Data Fig. 5c). Based on this, we hypothesized that 4-oxo-RA may circumvent the adverse cardiac effects of at-RA by selectively targeting HSCs in the BM (Fig. 4a). Indeed, and in sharp contrast to at-RA, in vivo administration of 4-oxo-RA had no significant impact on the myocardium (macrophages, monocytes, fibroblasts and endothelial cells), as shown by GSEA and DEGs (Fig. 4b and Extended Data Fig. 6a).

To explore the potential of 4-oxo-RA, in not only circumventing the adverse effects of at-RA but also favourably modulating the immune response after MI, we induced MI in mice followed by 4-oxo-RA (or vehicle) treatment for two consecutive days (Fig. 4c and Extended Data Fig. 6b).

In the acute phase of MI, we isolated BM HSPCs (Lineage^neg^, Sca1^pos^, cKit^pos^) to conduct scRNA-seq analysis. We grouped cells into three distinct major annotations based on molecular signatures: HSCs, MPPs and highly cycling MPPs (MPP-cyc) (Fig. 4d and Supplementary Table 5). Similar to our human data, we confirmed a significant reduction in the relative HSC numbers along with an expansion of MPP-cyc in vehicle-treated MI mice (Fig. 4e,f). Importantly, the 4-oxo-RA-treated group showed similar HSC and MPP-cyc percentages upon MI compared with sham control, suggesting mitigated HSC activation. GSEA showed that 4-oxo-RA treatment conserves HSC quiescence-related gene signatures and suppresses activated MPP signatures, underlining its potential to preserve HSC identity and counteract MI-induced HSC activation (Fig. 4g and Extended Data Fig. 6c). HSC lineage tracing using the *Fgd5*^*CreERT2*^ mouse model further demonstrated that 4-oxo-RA treatment significantly reduced the release of circulating dTomato^pos^ myeloid (CD11b^pos^) cells in PB during the 3-day time course (Extended Data Fig. 6d). We also observed reduced differentiation of HSCs towards the myeloid compartment (Fig. 4h,i). Of note, no significant effect was observed in lymphoid cells (Extended Data Fig. 6e). Mechanistically, 4-oxo-RA treatment maintained HSC quiescence after MI, as indicated by cell-cycle analysis and ex vivo scHSC division assays (Fig. 4j,k). In addition to enforcing quiescence, 4-oxo-RA treatment also maintained the self-renewal capacity of HSCs, as shown by in vitro CFU (acute phase) and in vivo HSC transplantation (chronic phase) assays (Fig. 4l–n and Extended Data Fig. 6f–h). Collectively, these results demonstrate that 4-oxo-RA can counter the functional decline of HSCs after MI.

HSCs migrate from the BM to the spleen in response to MI, where they contribute to extramedullary haematopoiesis^6^. We therefore conducted scRNA-seq analysis on splenic HSPCs and identified two major clusters in the spleen progenitor compartment: HSCs and MPPs (Fig. 4o and Supplementary Table 6). Mice treated with 4-oxo-RA showed an increased relative abundance of HSCs and a reduction in MPPs, suggesting dampened HSC activation in the spleen (Fig. 4p,q). This was further supported by GSEA results, which showed higher cell-cycle priming in the vehicle-treated compared with the 4-oxo-RA-treated group in spleen HSCs (Fig. 4r). These findings demonstrate that post-MI HSC activation occurs in the BM and spleen and can be mitigated by 4-oxo-RA treatment.

### 4-oxo-RA preserves long-term cardiac function upon MI

**Fig. 5:**
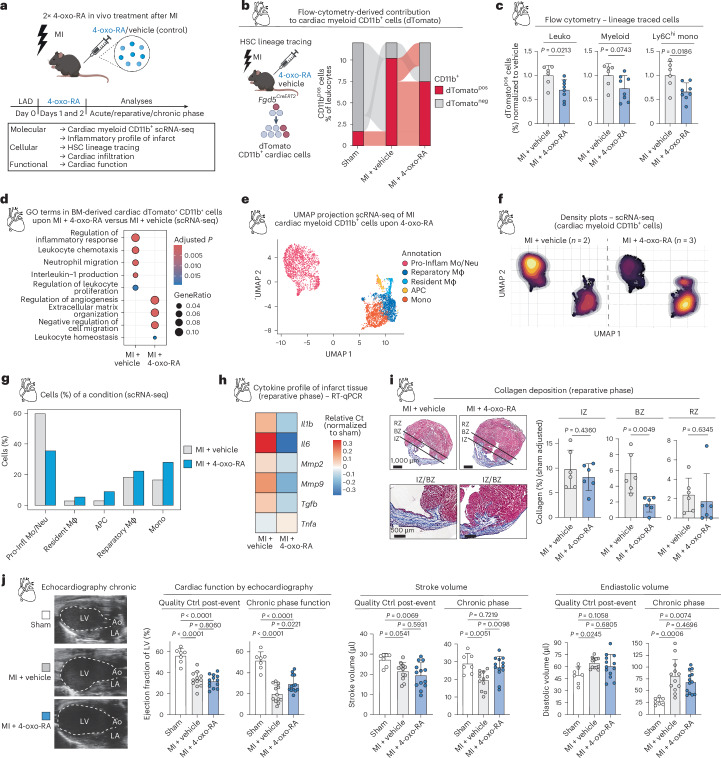
4-oxo-RA preserves long-term cardiac function via Rarβ upon MI. **a**, Experimental design to characterize HSC response after 4-oxo-RA in vivo treatment following MI. **b**, Alluvial plot of contribution from dTomato^pos^ and dTomato^neg^ cells to cardiac CD11b^pos^ myeloid cells in sham, vehicle and 4-oxo-RA upon MI based on flow cytometry. The *y* axis is reduced for visualization purposes. **c**, Flow-cytometry-based plots, illustrating the percentage of dTomato^pos^ cell frequencies for the vehicle and 4-oxo-RA condition in the myocardium upon MI. Cell frequencies are normalized to the vehicle condition. Data are presented as mean ± standard deviation. Ordinary two-way ANOVA. *n* = 6 vehicle; *n* = 8 4-oxo-RA. **d**, GO term enrichment of DEGs (top 200 log_2_FC, adjusted *P* value <0.05) in MI + vehicle compared with MI + 4-oxo-RA dTomato^pos^ myeloid cells based on scRNA-seq. **e**, UMAP plot of 4-oxo-RA mouse cardiac CD11b^pos^ cells scRNA-seq upon MI, based on projection on previously shown vehicle scRNA-seq. The colours indicate the predicted cell type annotation. *n* = 2 vehicle; *n* = 3 4-oxo-RA. **f**, UMAP density plots of vehicle and projected 4-oxo-RA mouse cardiac CD11b^pos^ cells scRNA-seq upon MI depicting relative cell abundance. **g**, Bar plot of quantified relative cluster abundance in MI + vehicle and projected MI + 4-oxo-RA mouse cardiac CD11b^pos^ cells scRNA-seq. **h**, Expression profiles of cytokines in cardiac cells in vehicle and 4-oxo-RA conditions in the reparative phase at day 10 after MI. Normalized mean relative to *Oaz1* expression and relative to sham surgery is shown. Values are relative to the average per gene. *n* ≥ 4 per gene. **i**, Masson’s Trichrome staining for collagen deposition in myocardial zones in MI + vehicle and MI + 4-oxo-RA. The lines highlight the separated areas in representative images. Two-tailed unpaired *t*-test. *n* = 6 per condition. RZ, remote zone; BZ, border zone; IZ, infarct zone. **j**, Cardiac functional assessment by echocardiography. Left: graphic representation of echocardiography during diastole. Ao, ascending aorta; LV, left ventricle; LA, left atrium. Right: quantification of echocardiographic parameters: EF, stroke volume and end-diastolic volume, comparing the quality control at day 1 with the chronic phase function at day 28 across treatment conditions after MI. Ordinary one-way ANOVA. *n* = 7 sham; *n* = 12 vehicle; *n* = 13 4-oxo-RA. In **b**–**g**, cells were isolated in the acute phase at day 3 after MI. Data are presented as mean ± standard deviation. *n* indicates the number of biological replicates per condition. For **b**, **c**, **h** and **j**, three or more independent experiments were performed. Source data

**Extended Data Fig. 7:**
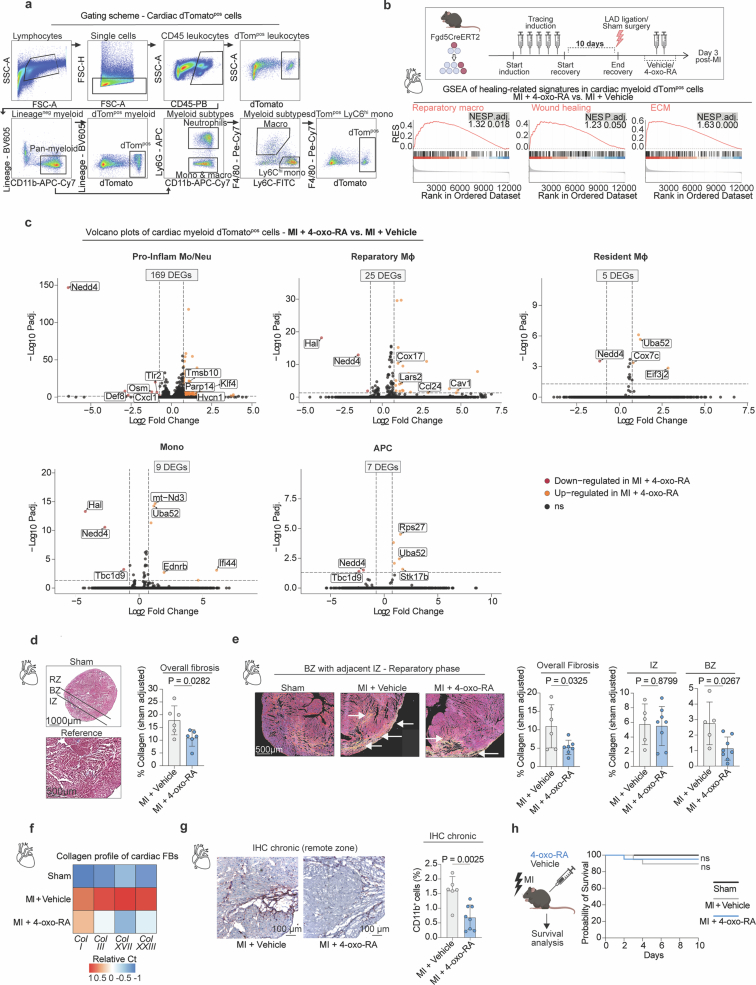
4-oxo-RA reduces cardiac inflammation and fibrosis upon MI. **a**, Representative gating scheme for flow cytometric analysis of dTomato^pos^ cells in the myocardium of Fgd5CreERT2 mice upon MI. **b**, GSEA profile of healing-related signatures in scRNA-seq of 4-oxo-RA and vehicle (DMSO) treated dTomato^pos^ cardiac myeloid cells upon MI. RES, Running enrichment score. **c**, Volcano plots of DEGs between 4-oxo-RA and vehicle (DMSO) treated dTomato^pos^ cardiac MI myeloid cells in each scRNA-seq subpopulation. Number of DEGs are depicted. **d**, Masson’s Trichrome staining for collagen deposition in whole myocardium in MI+vehicle and MI + 4-oxo-RA. A representative image of sham staining is depicted.Two-tailed unpaired t-test. n = 6. RZ, Remote zone; BZ, Border zone; IZ, Infarct zone. **e**, Picrosirius Red staining for collagen deposition in myocardium and separated zones in MI+vehicle and MI + 4-oxo-RA. Two-tailed unpaired t-test. n = 5-8. Representative images of staining are shown. Arrows highlight collagen depositions. **f**, qPCR expression profile of collagen genes in cardiac fibroblasts in sham, vehicle, and 4-oxo-RA treatment groups. Normalized mean relative to *Oaz1* expression is shown. n = 3-4. **g**, Left, Representative images and quantification of myocardium CD11b^pos^ IHC stainings of vehicle and 4-oxo-RA condition in the chronic phase at day 28 post MI. Scale bar, 100 µm. Right, Quantification of myocardium CD11b^pos^ IHC stainings of vehicle and 4-oxo-RA condition in the chronic phase at day 28 post MI. Two-tailed unpaired t-test. n = 10–11. **h**, Kaplan-Meier survival curves post MI and sham surgery. Mice were treated with either 4-oxo-RA or vehicle following MI and survival was monitored over a 10-day period. The survival probabilities for sham-operated, vehicle and 4-oxo-RA groups are depicted with ‘ns’ indicating non-significant differences between the groups. Mantel-Cox test. n = 35-41. In **c**, cardiac fibroblast were isolated 14 day after MI. In (**f-h**), cells were isolated in the acute phase at day three after MI. Data are presented as mean ± standard deviation. n indicates the number of biological replicates per condition or total number of human BM donors. Source data

HSC lineage tracing with the *Fgd5*^*CreERT2*^ model showed that 4-oxo-RA treatment reduced the proportion of dTomato^pos^ myeloid (CD11b^pos^) cells in the myocardium as measured by flow cytometry (Fig. 5a,b). Specifically, we observed a reduced contribution of HSCs into MI-induced neutrophils, monocytes and macrophages infiltrating the myocardium, including the inflammatory dTomato^pos^ Ly6C^hi^ monocyte population (Fig. 5c and Extended Data Fig. 7a). We next performed scRNA-seq analysis of HSC-traced myeloid (CD11b^pos^) cells in the myocardium and compared vehicle with 4-oxo-RA-treated mice using our refined short equilibrium tracing approach (Extended Data Fig. 7b and Supplementary Table 7). Overall comparison of cardiac dTomato^pos^ myeloid cells between 4-oxo-RA and vehicle-treated mice showed a downregulation of inflammation-related GO terms upon 4-oxo-RA treatment (Fig. 5d). We then projected 4-oxo-RA cells onto our MI scRNA-seq clusters (Figs. 2i and 5e) and found a relative enrichment of reparatory macrophages (Fig. 5f,g), which are defined by high levels of *Arg1* and enrichment of reparative processes such as extracellular matrix (ECM) and wound healing (Extended Data Fig. 3c–e). In line with this, we observed an upregulation of these healing-related terms in 4-oxo-RA treated dTomato^pos^ cells (Extended Data Fig. 7b). Pro-inflammatory monocytes and neutrophils were the most affected populations, showing a downregulation of proinflammatory genes such as *Cxcl1* upon 4-oxo-RA treatment (Extended Data Fig. 7c). Accordingly, we observed reduced expression of inflammatory cytokines in the overall infarct tissue during the reparative phase upon MI as shown by RT-qPCR (Fig. 5h). In addition, we quantified reduced collagen deposition, particularly in the remote zone of the heart, along with decreased expression of collagen-related genes in cardiac fibroblasts, collectively indicating a dampened scar formation upon 4-oxo-RA (Fig. 5i and Extended Data Fig. 7d–f). Immunohistochemistry staining showed reduced myeloid infiltration in the myocardium during the chronic phase (Extended Data Fig. 7g). Stroke volume and EF of the left ventricle were significantly preserved in the chronic phase after MI, demonstrating a significant improvement in heart function upon 4-oxo-RA treatment (Fig. 5j). Furthermore, 4-oxo-RA treatment also showed a slightly improved (non-significant) survival upon MI compared with vehicle-treated mice (Extended Data Fig. 7h).

These results provide strong evidence that 4-oxo-RA intervenes at the HSC level, altering their differentiation trajectory in the BM. This intervention preserves HSC functionality and effectively modulates the BM–heart myeloid axis. By reshaping both the quantitative and qualitative dynamics of myeloid infiltration into the cardiac tissue, 4-oxo-RA contributes to a suppressed inflammatory state and, thus, demonstrates promising therapeutic potential in enhancing post-MI cardiac function.

### Rarβ is indispensable for the beneficial effects of 4-oxo-RA

**Extended Data Fig. 8:**
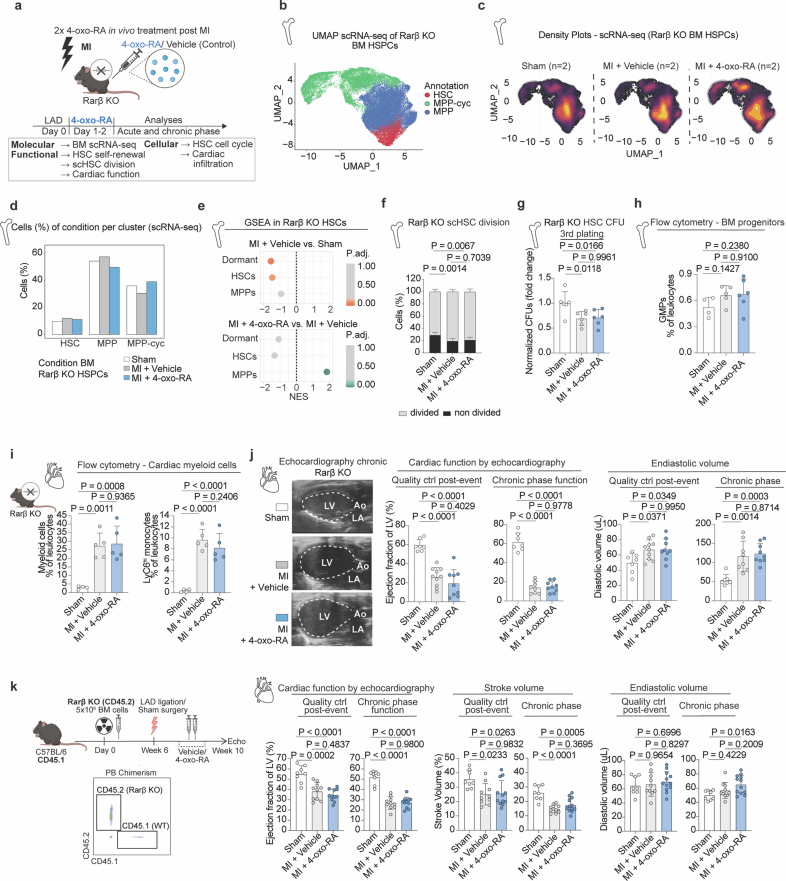
Rarβ is indispensable for 4-oxo-RA’s beneficial effects. **a**, Experimental design to characterize Rarβ KO HSC response after 4-oxo-RA *in vivo* treatment following MI. **b**, UMAP plot of scRNA-seq on BM HSPC cells isolated from Rarβ KO mice that received *in vivo* treatment with vehicle or 4-oxo-RA post MI, as well as from Rarβ KO mice undergoing sham surgery. n = 2. **c**, UMAP density plots of each condition in BM Rarβ KO HSPC scRNA-seq depicting relative cell abundance. **d**, Bar plot of quantified relative cluster abundance in each condition based on scRNA-seq data of BM Rarβ KO HSPC cells. **e**, GSEA of published mouse HSPC signatures in the pairwise comparisons between MI+vehicle vs. sham HSC cluster and MI + 4-oxo-RA vs. MI+vehicle HSC cluster based on Rarβ KO scRNA-seq. **f**, ScHSC division assay after 48 h in sham, MI+vehicle and MI + 4-oxo-RA Rarβ KO HSCs. The percentage of cells is shown. Depicted p values correspond to the percentage of non-divided cells. Statistics denote comparisons between vehicle or 4-oxo-RA condition and the sham condition. Ordinary two-way ANOVA. n = 5–6. **g**, 1^st^, 2^nd^ and 3^rd^ plating of Rarβ KO HSC CFU assay comparing sham, MI+vehicle and MI + 4-oxo-RA condition. Ordinary two-way ANOVA. n = 6. **h** and **i**, Flow cytometry-based analysis of emergency hematopoiesis in Rarβ KO mice during the acute phase at day three post MI. Percentage of leukocyte cell frequencies is depicted in BM and myocardium. Ordinary two-way ANOVA. BM and myocardium, n = 4–6. **j**, Left, Representative echocardiographic images showing left ventricular (LV) morphology during diastole in sham, MI with vehicle, and MI with 4-oxo-RA treatment groups. Ao, ascending aorta; LA, left atrium; LV, left ventricle. Right, Analysis of ejection fraction and endiastolic volume at day one post MI (as quality control) and in the chronic phase at day 28 post MI. Ordinary one-way ANOVA. n = 7-10. **k**, Left, Schematic design of chimera experiments to characterize the transplantation of BM Rarβ-KO cells into wildtype mice followed by MI and 4-oxo-RA in viv*o* treatment. Right, Analysis of ejection fraction, stroke volume and endiastolic volume in sham, MI with vehicle, and MI with 4-oxo-RA treatment groups at day one post MI (as quality control) and in the chronic phase at day 28 post MI. Ordinary one-way ANOVA. n = 8-11. In **c**, cardiac fibroblast were isolated 14 day after MI. In (**a-e**), cells were isolated in the acute phase at day three after MI. Data are presented as mean ± standard deviation. n indicates the number of biological replicates per condition or total number of human BM donors. For (**b-e**), two or more independent experiments were performed. Source data

To mechanistically investigate the role of Rarβ in facilitating the beneficial effects of 4-oxo-RA on myocardial outcomes, we subjected Rarβ-knockout (KO) mice to MI followed by 2-day 4-oxo-RA treatment (Extended Data Fig. 8a). In the absence of Rarβ, 4-oxo-RA treatment did not maintain HSC function and neither mitigated the downstream myeloid response in the BM and myocardium as shown by scRNA-seq analysis, scHSC division, CFU assays and flow cytometry analysis (Extended Data Fig. 8b–i and Supplementary Table 8). Ultimately, 4-oxo-RA proved ineffective in preserving cardiac function in the chronic phase of MI in Rarβ-KO mice (Extended Data Fig. 8j). Similarly, we transplanted Rarβ-KO BM cells into wild-type recipient mice, which then underwent MI via LAD ligation. In these Rarβ-KO chimeras, treatment with 4-oxo-RA showed no improvement in the cardiac function, highlighting the lack of a beneficial effect when Rarβ is specifically deleted in the BM (Extended Data Fig. 8k). In conclusion, 4-oxo-RA mediates its beneficial effect on HSC protection and cardiac remodelling through Rarβ at the BM level.

### RA metabolites restore the function of human HSPCs impaired by MI

**Fig. 6:**
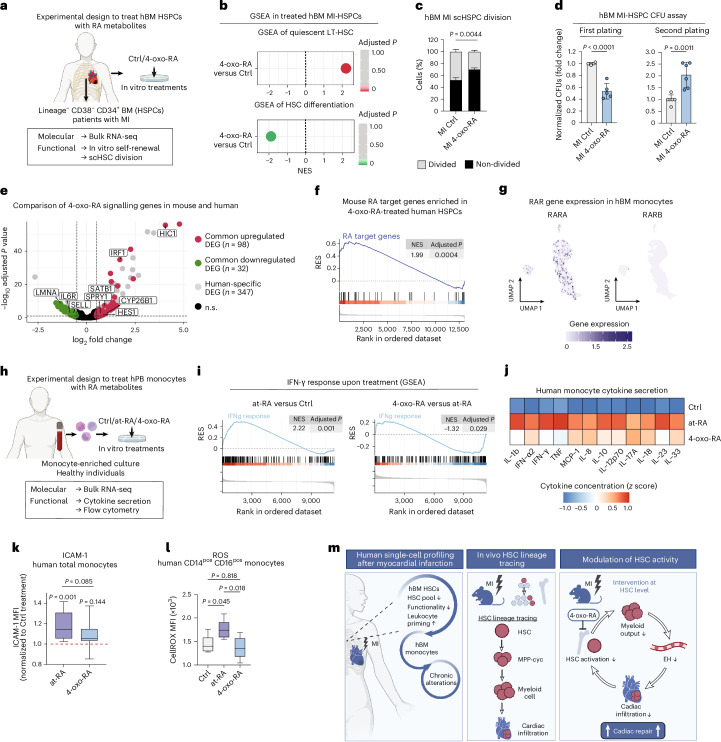
4-oxo-RA restores human HSC function after MI and mitigates at-RA-induced human monocyte inflammation. **a**, Experimental design to characterize human sternal BM HSPCs of MI and control (Ctrl) donors after in vitro treatment with 4-oxo-RA. **b**, GSEA of published human HSPC signatures in human HSPCs upon in vitro culture with 4-oxo-RA treatment versus control (DMSO) from patients with MI based on population RNA-seq. *n* = 2 per condition. **c**, scHSPC division assay after 48 h in MI-HSPCs after in vitro treatment with 4-oxo-RA or control (DMSO). The percentage of cells is shown. Depicted *P* values correspond to the percentage of non-divided cells. Ordinary two-way ANOVA. *n* = 3 per condition. **d**, First and second plating of human MI-HSPC CFU assay after in vitro treatment with 4-oxo-RA or control (DMSO). Two-tailed unpaired *t*-test. *n* = 6 per condition. **e**, Volcano plots of DEGs between 4-oxo-RA and DMSO (Ctrl) treatment in human BM HSPCs from healthy donors. DEGs that are common in previously published 4-oxo-RA and DMSO (Ctrl) treatment in mouse BM HSCs (log_2_FC threshold 0.5, adjusted *P* value <0.1) are coloured in red (upregulated) or green (downregulated). In total, 94 out of 331 upregulated human genes were conserved in mouse (28%), while 32 out of 142 genes (23%) were downregulated in both species. Important genes are annotated. **f**, GSEA of mouse RA direct target gene list in human HSPCs upon 4-oxo-RA treatment versus control (DMSO) from healthy donors based on population RNA-seq. **g**, UMAP plots showing expression levels of RARA and RARB genes in human BM monocyte scRNA-seq. **h**, Experimental design to characterize human PB monocytes (monocyte-enriched culture) of healthy donors after in vitro treatment with control (DMSO), at-RA and 4-oxo-RA. *n* = 4 per condition. **i**, GSEA of IFN-γ response signature in human PB monocytes upon at-RA treatment versus control (DMSO) and 4-oxo-RA versus at-RA treatments from healthy donors based on population RNA-seq. **j**, Cytokine secretion assay in supernatant of human PB monocyte cultures from healthy donors upon control (DMSO), at-RA and 4-oxo-RA treatments. **k**, ICAM-1 expression in human PB monocytes (CD14^pos^ and/or CD16^pos^ monocytes) from healthy donors upon control (DMSO), at-RA and 4-oxo-RA treatments. MFI, mean fluorescence intensity. The central line denotes the median; the boxes denote lower and upper quartiles (Q1 and Q3, respectively); the whiskers represent the minimum and maximum values. *n* = 12–13. **l**, Reactive oxygen species (ROS) levels assessed by CellROX in human PB intermediate monocytes (CD14^pos^ CD16^pos^) from healthy donors upon control (DMSO), at-RA and 4-oxo-RA treatments. MFI, mean fluorescence intensity. The central line denotes the median; the boxes denote lower and upper quartiles (Q1 and Q3, respectively); the whiskers represent the minimum and maximum values. *n* = 5–6. **m**, Schematic representation of main findings. Data are presented as mean ± standard deviation. *n* indicates the number of biological replicates (mice or human BM donors) per condition. For **c**, **d**, **k** and **l**, three or more independent experiments were performed. Source data

**Extended Data Fig. 9:**
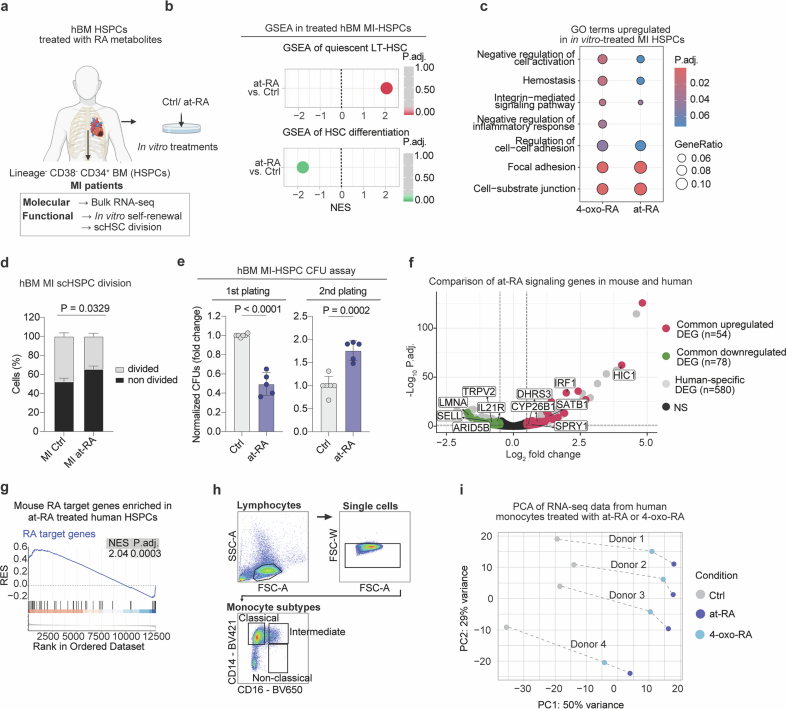
at-RA modulates human MI-HSCs. **a**, Left, Experimental design to characterize human sternal BM HSPCs of MI and Ctrl (control) donors after *in vitro* treatment with at-RA. Right, GSEA of published human HSPC signatures in human HSPCs upon *in vitro* culture with at-RA treatment vs. control (DMSO) from MI patients based on population RNA-seq. n = 2–4. **b**, GSEA of published human HSPC signatures in human HSPCs upon *in vitro* culture with at-RA treatment vs. control (DMSO) from MI patients based on population RNA-seq. n = 2–4. **c**, GO terms enrichment of upregulated DEGs (log2FC threshold = 0, P.adj < 0.1) in human MI-HSPCs with at-RA or 4-oxo-RA treatment vs. control (DMSO). **d**, ScHSPC division assay after 48 h in MI HSPCs after *in vitro* treatment with at-RA or control (DMSO). The percentage of cells is shown. Depicted p values correspond to the percentage of non-divided cells. Ordinary two-way ANOVA. n = 3. **e**, 1^st^ and 2^nd^ plating of human MI HSPC CFU assay after *in vitro* treatment with at-RA or control (DMSO). Unpaired t-test. n = 6. **f**, Volcano plots of DEGs between at-RA and DMSO (Ctrl) treatment in human BM HSPCs from healthy donors. DEGs that are common in previously published at-RA and DMSO (Ctrl) treatment in mouse BM HSCs (log2FC threshold = 0.5, P.adj < 0.1) are coloured in red (upregulated) or green (downregulated). 54 out of 313 upregulated human genes were conserved in mouse (17 %), while 78 out of 399 genes (20 %) were downregulated in both species. Important genes are annotated. **g**, GSEA of mouse RA direct target gene list in human HSPCs upon at-RA treatment vs. control (DMSO) from healthy donors based on population RNA-seq. **h**, Representative gating scheme for flow cytometric analysis of monocyte subtypes in PB of healthy donors. **i**, PCA of human PB monocytes from healthy donors upon control (DMSO), at-RA or 4-oxo-RA treatments based on the top 500 most variable genes in RNA-seq. n = 4. Data are presented as mean ± standard deviation. n indicates the number of biological replicates per condition or total number of human BM donors. For (**d** and **e**), three or more independent experiments were performed. Source data

Considering that modulating HSC-dependent inflammatory myelopoiesis improves cardiac function in the mouse, we next addressed whether RA metabolites can restore human HSCs after MI-induced impairment. To do this, we isolated human HSPCs from patients with MI and treated them with the RA metabolites 4-oxo-RA and at-RA (Fig. 6a and Extended Data Fig. 9a).

Using population RNA-seq and GSEA, we observed a significant enrichment of gene signatures linked with human quiescent HSCs, indicating a preserved HSC identity upon culture (Fig. 6b and Extended Data Fig. 9b). This was accompanied by the upregulation of processes related to cell adhesion and negative regulation of cell activation and inflammatory response (Extended Data Fig. 9c). Notably, treatment with RA metabolites also downregulated differentiation signatures, indicating their role in suppressing human HSC differentiation in culture. Functionally, both metabolites attenuated HSPC proliferation in single-cell division assays (Fig. 6c and Extended Data Fig. 9d). Post-treatment CFU assays demonstrated that both RA metabolites maintained in vitro self-renewal capacity (Fig. 6d and Extended Data Fig. 9e). To further investigate the translatability of RA signalling to human, we assessed the differential expression of the human dataset and our previously published mouse data upon RA treatment^20^ (Fig. 3c). We found a notable overlap of upregulated genes related to HSC quiescence and of downregulated genes associated with cell cycle and differentiation upon RA treatment in both species^36–41^ (Fig. 6e, Extended Data Fig. 9f and Supplementary Table 9). In addition, direct target genes of RA in mouse^20^ were significantly enriched in human RA-treated HSPCs, indicating a conserved RA signalling among both species (Fig. 6f, Extended Data Fig. 9g and Supplementary Table 9).

Finally, we aimed to investigate the effect of retinoids in human monocytes. Indeed, human BM monocytes express RARA and not RARB, in line with the mouse data (Fig. 6g and Fig. 3o). Thus, we isolated human PB monocytes from healthy donors and treated them with both RA metabolites. RNA-seq analysis revealed an upregulation of IFN-γ response after at-RA treatment, which was attenuated by 4-oxo-RA (Fig. 6h,i and Extended Data Fig. 9h,i). This regulatory pattern was consistent across various inflammatory cytokines, as determined by a cytokine secretion assay (Fig. 6j). Furthermore, 4-oxo-RA treatment resembled control-treated monocytes, showing low expression of ICAM-1, a key molecule in leukocyte recruitment^42^, and reduced reactive oxygen species levels that are associated with monocyte activation^43,44^, compared with at-RA treatment (Fig. 6k,l).

In conclusion, 4-oxo-RA effectively counteracted the impairment of human HSC function after MI and bypassed the inflammatory effects of at-RA on human monocytes. These findings underscore its potential in therapeutic interventions at the level of human HSCs upon MI to prevent excess myelopoiesis in patients.

## Discussion

In summary, we show that, upon MI, 4-oxo-RA treatment enforces HSC quiescence, thereby mitigating EH and ultimately preserving cardiac function (Fig. 6m). Upon 4-oxo-RA treatment after MI, we observed (1) dampened HSC activation; (2) a reduction in HSC-derived inflammatory myeloid cells infiltrating the heart; and (3) no beneficial effects on HSCs and cardiac function in the Rarβ-KO mice and wild-type mice transplanted with Rarβ-KO BM. Furthermore, we found (4) specific expression of the high affinity 4-oxo-RA receptor *Rarb* in BM HSCs compared with downstream haematopoietic cell populations, BM niche cells and cardiac tissue in homeostatic conditions and after MI. Finally, (5) we show absence of transcriptional changes in cardiac cell populations upon 4-oxo-RA treatment, confirming a high therapeutic specificity to HSCs.

Our study bridges a critical translational gap in the BM–heart axis and demonstrates HSC activation and loss of functionality in human patients after MI, consistent with previous studies performed in mice^24^. These findings highlight a detrimental impact on the BM HSC pool that is independent of chronic inflammation observed in heart failure patients^45,46^. We show that patients with MI display a myeloid-priming signature at the HSPC level that is accompanied by a persistent inflammatory state and distinct temporal distribution of monocytes in the BM. Indeed, it has been reported that elevated levels of circulating monocytes correlate with worsened ventricular remodelling in patients with MI^47–49^. This implies that reduced monocytes could be beneficial for cardiac recovery and could thus dampen the risk of subsequent ischaemic events. Due to the lack of tracing tools, we and others could not provide any evidence of a direct contribution from BM MI-activated HSCs to cardiac infiltrating monocytes^22–24^. Indeed, the role of HSCs in producing differentiated cells upon EH has been recently challenged^25–27^. Using an HSC mouse lineage-tracing approach^31,50^, our data suggest the rapid contribution of BM HSCs to myeloid cell production in the acute phase after MI. Our study delineates a possible differentiation pathway of HSCs, progressing from MPPs to MyP in the BM, ultimately leading to the presence of inflammatory myeloid cells within the infarcted heart. It is important to note that our tool cannot rule out partial contributions from other compartments. Some labelling activity in MPPs or other haematopoietic compartments downstream of HSCs cannot be completely excluded, potentially confounding the specificity of HSC lineage tracing. Despite this limitation, the Fgd5 allele is the most specific model that still allowed us to trace enough cells into the heart for profiling, which confirmed the inflammatory properties of HSC-derived myelopoiesis in the myocardium.

Targeting systemic inflammation has been suggested as a potential MI treatment. We previously showed that systemic inhibition of IL-1β, known to be produced by cardiac myeloid cells, reduced the myeloid cells and general cardiac inflammatory response^14^. However, this reduction occurs in a non-selective manner with multiple unknown effects in other organs. This might explain why human clinical studies targeting overall inflammation have led to inconsistent outcomes^10–13^. We therefore propose to target the root of the inflammatory events by modulating specifically HSC activity and, thus, downstream myeloid production, limiting disease progression.

We previously showed that active metabolites of vitamin A play a crucial role in modulating in vivo mouse HSC quiescence^19,20^. Here, we reveal the potential of at-RA, extensively used in different clinical applications, in inhibiting human HSC activation and mitigating emergency myelopoiesis. However, we also observe a pro-inflammatory response in cardiac myeloid cells that may counteract cardiac remodelling benefits. This may be due to at-RA’s non-selective binding within RA receptors, leading to diverse cellular responses^51–53^. In line with our observations, previous studies have reported contradictory roles for at-RA in cardiac repair after MI. Some studies suggest cardioprotective effects of at-RA, such as the reduction of cardiac hypertrophy^54^ and anti-apoptotic effects after MI^55,56^. However, other studies indicate adverse effects on MI outcomes^57,58^. Our study underlines the broad impact of at-RA upon systemic administration and might contribute to clarifying these conflicting results. In addition, our findings highlight the importance of evaluating at-RA treatment in post-MI patients.

By contrast, 4-oxo-RA exhibits higher binding specificity for Rarβ (ref. ^59^) that is exclusively expressed in HSCs^19,60–62^ among all cell populations present in BM and not in cardiac tissue^63^. Here, we that 4-oxo-RA treatment specifically attenuates MI-HSC activation, thereby reducing myelopoiesis and off-target effects on myeloid cells. In the myocardium, we observe a reduced monocyte recruitment and an enrichment of reparatory-type macrophages. A decrease in cardiac inflammation may also establish a microenvironment that leads to a differential programming of infiltrating monocytes towards a more reparative state. Taken together, this may contribute to the reduction of fibrosis and observed improvement of cardiac function upon 4-oxo-RA treatment. Future studies should also explore whether 4-oxo-RA treatment impacts additional functional outcomes to cardiovascular health, such as exercise tolerance, which may further substantiate the therapeutic benefits after MI.

## Methods

### Mouse models

Mice used in this study were bred in-house at the Max Planck Institute of Immunology and Epigenetics (MPI-IE) and housed in individually ventilated cages, with the exception of C57Bl6J wild types with induced MI, which were purchased from Janvier and Charles River. All mice with induced MI were conventionally housed at the Freiburg Center for Experimental Models and Transgenic Service. Only female mice aged 6–12 weeks were used. Euthanasia followed German guidelines using cervical dislocation or CO_2_ gas inhalation. Animal procedures followed approved protocols by German authorities and Regierungspräsidium Freiburg, under §4 (3) of the German Animal Protection Act, with animal protocol numbers 35-9185.81/G-19/32, 35-9185.81/G-19/112, 35-9185.81/G21/063 and 35-9185.81/G-22/102. Lineage tracing experiments were performed under protocol 23-048-PIL approved by the Comitè Ètic d'Experimentació Animal (CEEA) committee of Parc Científic de Barcelona.

### Transplantation models

Female mice, either B6Ly5.1 (CD45.1) or C57BL/6J × B6Ly5.1 (CD45.1/2), were used as recipients for transplantation experiments and/or as supportive or competitive donors. Recipient mice were aged 6–12 weeks. Supportive or competitive donors were age-matched to their respective control group.

### Rarβ-KO mouse model

Mice lacking the *Rarb* gene were purchased from The Jackson Laboratory (Jax Stock Rarβtm1Vgi/HsvJ; stock no. 022999). Breeding was conducted at MPI-IE. Experimental procedures involved exclusively female mice aged 6–12 weeks.

### NBSGW

Female NBSGW mice (Jax Stock NOD.Cg-*Kit*^*W-41J*^ *Tyr*^+^ *Prkdc*^*scid*^ *Il2rg*^*tm1Wjl*^/ThomJ; stock no. 026622), aged 6–12 weeks, were used as transplantation recipients for human HSPCs (Lineage^neg^, CD34^pos^).

### Fgd5 lineage tracing (*Fgd5*^*CreERT2*^ × *Rosa26*^*LSL-tdTomato*^)

*Fgd5*^*CreERT2*^ mice express a tamoxifen-inducible Cre recombinase and green fluorescent protein (ZsGreen) in the Fgd5 locus being active in HSCs (C57BL/6N-Fgd5tm3(cre/ERT2)Djr/J; stock no. 027789). This model was crossed with *Rosa26*^*LSL-tdTomato*^ mice (B6.Cg-*Gt(ROSA)26Sortm14(CAG-tdTomato)Hze*/J; stock no. 007914). Expression of red fluorescent protein (tdTomato) is controlled by a loxP-flanked STOP cassette. Upon Cre-mediated recombination, robust tdTomato fluorescence is observed, allowing lineage tracing from HSCs into downstream compartments^32^.

For labelling induction, mice received intraperitoneal (i.p.) injections of tamoxifen dissolved in oil at 75 mg kg^−1^ body weight once daily for 5 days, between 5 and 10 weeks of age. After a 4-week period to verify successful labelling (using platelets as the reference), MI surgery was performed on mice, followed by treatment with either 4-oxo-RA or a control vehicle, as detailed in ‘RA/vitamin A in vivo treatments after MI’. The mice used in the study were bred at IRB Barcelona under protocol 22-001-ARF.

Labelling propagation analysis was performed using the HSC population as the top reference compartment (CD48^neg^, CD150^pos^, LSK) and the MPP (CD48^pos^, CD150^neg^, LSK) and MyP (LS-K) populations as downstream compartments. Data were analysed using a mixed-effects model using the percentage of tdTomato as the dependent variable and the population and the treatment as independent variables.

1. Model equation:
  $${Y_{ij}}={{{\beta }}}_{0}+{{{\beta }}}_{1}{{{X}}}_{1,i,\,j}+{{{\beta }}}_{2}{{{X}}}_{2,i,\,j}+\ldots +{{\beta_nX_{n,i,\,j}}}+{{u_i}}+{{\varepsilon }}{{_{ij}}}$$
  *Y*_*ij*_ is the dependent variable (for example, percentage of tdTomato) for the *i*th mouse in the *j*th condition.
  *β*_0_ is the intercept.
  *β*_1_, *β*_2_,…, *β*_*n*_ are the fixed-effect coefficients for each independent variable *X*_1_, *X*_2_,…, *X*_*n*_ (for example, treatment conditions such as baseline, sham and MI, and populations).
  *u*_*i*_ is the random effect for the *i*th mouse, capturing individual variability.
  *ε*_*ij*_ is the random error term for the *i*th mouse in the *j*th condition.
2. Random effects:
  $${{u_i}}\approx {{N}}\left(0,\,{\rm{\sigma}}^{2}_{u}\right),$$
  where *N*(0, *σ*^2^_*u*_) denotes a normal distribution with mean 0 and variance *σ*^2^_*u*_.
3. Error term:

${{\varepsilon }}{{_{ij}}}\approx {{N}}(0,\,{{{\sigma }}}^{2}_{\varepsilon }),$ where *N*(0, *σ*^2^_*ε*_) denotes a normal distribution with mean 0 and variance *σ*^2^_*ε*_.

### Experimental in vivo mouse experiments

#### Operation of LAD artery for MI surgery

MI was induced in female C57BL/6J or Rarβ-KO mice aged approximately 6–12 weeks through permanent occlusion of the LAD artery ligation. Anaesthesia was induced by i.p. injection of 100 mg kg^−1^ ketamine (Zoetis) and 10 mg kg^−1^ xylazine (Bayer Vital). Analgesia was initiated around 30 min before surgery through subcutaneous injection of 0.1 mg kg^−1^ buprenorphine. To account for perioperative dehydration resulting from blood loss and perspiration, 20 ml kg^−1^ of isotonic 5% glucose injection solution (Glucosteril; Fresenius Kabi Deutschland GmbH) in 0.9% NaCl (9 mg ml^−1^) was administered by i.p. injection. A small animal respirator (MiniVent ventilator for mice model 845; Hugo Sachs Elektronik) was used with ventilation set at a positive end-inspiratory pressure of 5 mbar, a respiratory rate of 110 breaths min^−1^ and an inspiration/expiration ratio of 1/1.5. Throughout the procedure, oxygen saturation, heart rate and respiratory rate were monitored using the MouseOX system (Starr Life Sciences). Anaesthesia was sustained by the addition of 0.5–2% isoflurane (AbbVie) during surgery. After right lateral positioning of the animal, shaving and skin disinfection, a left lateral thoracotomy was conducted between the third and fourth rib. The pericardium was opened to identify the LAD coronary artery. Permanent LAD ligation was executed in the proximal middle third of the LAD, utilizing an 8–0 prolene suture (Ethicon). Upon evacuating the pneumothorax, closure of chest and skin wounds was accomplished with a 5–0 or 6–0 prolene suture (Ethicon).

#### MI experiments in RarB-KO chimeras

Female wild-type C57BL/6 (CD45.1) recipient mice were subjected to cumulative, lethal irradiation with a total dose of 9.5 Gy. Haematopoietic reconstitution was achieved by transplanting 5 million BM cells from Rarβ-KO donor mice via tail vein injection. Four weeks after transplantation, PB was collected via puncture of the facial vein to assess chimerism and confirm complete haematopoietic reconstitution of the wild-type C57BL/6 (CD45.1) BM with that from Rarβ-KO mice. LAD ligation or sham surgery was induced as described and at least 6 weeks after transplantation. During the chronic phase of myocardial healing (at day 28), cardiac function was evaluated by echocardiography.

#### RA/vitamin A in vivo treatments after MI

C57BL/6J or Rarβ-KO mice were intraperitoneally injected on the first and second days after MI surgery with either 30 mg kg^−1^ body weight at-RA (Sigma-Aldrich), 30 mg kg^−1^ body weight 4-oxo-RA (Sigma-Aldrich) or the corresponding amount of DMSO in phosphate-buffered saline (PBS). Mice were euthanized during the acute phase of MI (days 2 and 3), the reparative phase (day 7) and the chronic phase of MI (days 21 and 28). After euthanasia, HSCs were isolated and underwent further analysis, including assessments of cell cycle, single-cell division assays and CFU assays (as detailed below).

#### Echocardiography

Echocardiography was conducted following the methodology previously published in ref. ^64^. Parameters including left ventricular EF, end-systolic and end-diastolic volume and stroke volume were evaluated.

### Downstream characterization of mouse models

#### Histology

For immunohistochemistry analysis, hearts were collected and embedded in OCT compound (Sakura Finetek), then snap-frozen on dry ice. Sections of 5 µm thickness were stained using an anti-CD11b antibody (BD Pharmingen, cat. no. 553308, 1:250). Staining was followed by biotinylated secondary antibody (Vector Labs, cat. no. BA-4001, 1:200). We used the VECTASTAIN Elite ABC HRP kit and ImmPACT AMEC Red Peroxidase (HRP) substrate (Vector Laboratories) for colour development. Heart sections from Fgd5CreERT2 mice were stained with 4′,6-diamidino-2-phenylindole (DAPI), and analysis was conducted using the particle analyser feature in ImageJ. dTomato^pos^ counts were normalized to DAPI^pos^ counts for quantification.

#### Image analyses after MI

Tissue sections were imaged and subsequently analysed using Fiji/ImageJ software. To assess collagen deposition in different cardiac regions, a colour deconvolution algorithm was applied to separate specific staining components. The deconvoluted images were then thresholded to segment areas of interest on the basis of pixel intensity values corresponding to collagen.

Regions of interest corresponding to the infarct zone, border zone and remote zone of the heart were manually defined and adjusted for each sample. These regions of interest were used to measure collagen content within the designated zones. Thresholding was performed to ensure accurate quantification of collagen-positive areas. All measurements were taken across predefined regions and were standardized for comparison across samples.

In the case of dTomato^pos^ cell analysis in cardiac tissue, sections were also stained with DAPI to visualize nuclei. This allowed the normalization of dTomato-positive cell counts relative to the total number of DAPI^pos^ cells, providing accurate assessment of cell abundance within the tissue.

#### Flow-cytometric heart analysis after MI

After euthanasia of the mice, hearts were extracted to analyse the infarcted myocardium. Infarcted myocardial tissue was excised, minced and subjected to enzymatic digestion using a mixture of collagenase I (450 U ml^−1^), collagenase XI (125 U ml^−1^), DNase I (26 U ml^−1^) and hyaluronidase (60 U ml^−1^) (all obtained from Sigma-Aldrich). Enzymatic reaction was incubated at a temperature of 37 °C and a rotation speed of 600 rpm for a duration of 1 h and subsequently stopped by the addition of 30 ml of fluorescence-activated cell sorting (FACS) buffer consisting of PBS containing 0.5% bovine serum albumin (BSA) and 1% foetal bovine serum.

Cardiac cell suspension was stained using the following antibodies (all obtained from BioLegend unless stated otherwise): Lineage-BV605 (CD19 (cat. no. 115539, 1:500), CD90 (cat. no. 105343, 1:500), CD4 (cat. no. 100547, 1:500), CD8a (cat. no. 100743, 1:500), NK1.1 (cat. no. 108739, 1:500), Ter119 (cat. no. 116239, 1:500), CD49b (BD Pharmingen, cat. no. 740363, 1:500)), Ly6C-FITC (cat. no. 128006, 1:500), CD115-BV711 (cat. no. 135515, 1:500), Ly6G-APC (cat. no. 127614, 1:500), CD11b-APC-Cy7 (cat. no. 101226, 1:500), CD45.2-PB (cat. no. 109820, 1:500) and F4/80-Pe-Cy7 (cat. no. 123113, 1:500).

#### Flow-cytometric blood, spleen and whole BM analysis after MI

After the extraction of venous blood via tail vein puncture, mice were euthanized to collect spleen, femurs, tibiae and pelvis for BM analysis.

Venous blood was collected using 5 mM EDTA (Sigma-Aldrich). The spleen was filtered through a 40-µm cell strainer to create a single-cell suspension. The BM was crushed and then filtered through a 40-µm cell strainer to create a single-cell suspension. Cell suspensions were treated with red blood cell lysis buffer (BioLegend) for subsequent staining.

Spleen, blood and whole BM were stained using the following antibodies (all obtained from BioLegend): Ly6C-FITC (cat. no. 128006, 1:500), CD115-BV711 (cat. no. 135515, 1:500), B220-BV650 (cat. no. 103241, 1:500), CD3-PerCP-Cy5.5 (cat. no. 100218, 1:500), Ly6G-APC (cat. no. 127614, 1:500), CD11b-APC-Cy7 (cat. no. 101226, 1:1,000) and CD45.2-PB (cat. no. 109820, 1:500). In addition, in a separate staining procedure, spleen, blood and BM were stained using the following antibodies: Lineage-BV650 (Gr1 (cat. no. 108442, 1:1,000), CD11b (cat. no. 101259, 1:1,000), B220 (cat. no. 103241, 1:500), Ter119 (cat. no. 116235, 1:500), CD4 (cat. no. 563232, 1:1,000), CD8a (cat. no. 100722, 1:1,000)), cKit-BV711 (cat. no. 105835, 1:1,000), Sca1-APC-Cy7 (cat. no. 108126, 1:500), CD150-PeCy5 (cat. no. 115912, 1:500), CD48-BV421 (cat. no. 103428, 1:1,000), CD16/32-APC (cat. no. 101326, 1:1,000), CD34-FITC (BD Biosciences, cat. no. 553733, 1:50) and CD127-Pe-Cy7 (cat. no. 135013, 1:200).

#### Enrichment of mouse HSPCs and isolation of HSCs

Murine BM cells were isolated from the femur, tibia, hip bone and vertebrae by gentle crushing with mortar and pestle in PBS. Red blood cell lysis was performed with ACK Lysing Buffer (Thermo Fisher Scientific) for 5 min at room temperature. Dynabeads Untouched Mouse CD4 Cells Kit (Invitrogen) was used for lineage negative enrichment according to the manufacturer’s protocol. In brief, the BM was stained with 1:4 dilution of the Lineage Cocktail for 30–60 min at 4 °C on a rotating wheel. Labelled cells were then incubated for 20 min with 400 µl of washed Dynabeads coated with polyclonal sheep anti-rat IgG per sample. Depletion of lineage cells was performed using a magnet. Lineage-depleted BM cells were stained with lineage markers using the following antibodies (all obtained from BioLegend unless stated otherwise): Lineage-BV650 (Gr1 (cat. no. 108442, 1:1,000), CD11b (cat. no. 101259, 1:1,000), B220 (cat. no. 103222, 1:500), Ter119 (cat. no. 116235, 1:500), CD4 (cat. no. 563232, 1:1,000), CD8a (cat. no. 100722, 1:1,000), ckit-BV711 (cat. no. 105835, 1:1,000), Sca1 (cat. no. 108126, 1:500), CD150-PE/Cy5 (cat. no. 115912, 1:500), CD48-PE/Cy7 (cat. no. 103424, 1:500) and CD34-FITC (BD Biosciences, cat. no. 553733, 1:50). Sorting was then performed using a FACS Aria II, III or FACSymphony (Becton Dickinson). Subsequently, cells were collected into ice-cold PBS for reconstitution assays, Complete Stem Cell Medium (StemPro-34 SFM, Life Technologies, supplemented with 50 ng ml^−1^ stem cell factor (SCF), 25 ng ml^−1^ thrombopoietin (TPO), 30 ng ml^−1^ Flt3-Ligand (all from Preprotech), 100 µg ml^−1^ penicillin–streptomycin and 2 mM l-glutamine (both from Gibco)) for experiments for in vitro culture, PBS with 2% BSA for scRNA-seq and RNA lysis buffer (Arcturus PicoPure RNA Isolation Kit (Applied Biosystems)) for population RNA-seq and stored at −80 °C.

#### Cell-cycle analysis

After lineage depletion, erythrocyte-lysed BM was stained for HSC markers (Lineage^neg^, cKit^pos^, Sca-1^pos^, CD150^pos^, CD48^neg^, CD34^neg^) as specified above. Subsequently, cells were fixed for 10 min at 4 °C using BD Cytofix/Cytoperm Buffer (Becton Dickinson and Company) and intracellular Ki-67-PE (Invitrogen, cat. no. 12-5698-80, 1:500) staining was performed using PermWash solution for at least 45 min at 4 °C (Becton Dickinson and Company). Before proceeding with flow cytometry analysis, the cells were stained with DAPI (Sigma-Aldrich) at room temperature for a minimum of 20 min.

#### Mouse single-cell division assay

Individual HSCs (Lineage^neg^, cKit^pos^, Sca-1^pos^, CD150^pos^, CD48^neg^, CD34^neg^) were sorted into 72-well Terasaki plates (Greiner Bio-One) containing Complete Stem Cell Medium (as specified above). After a 48-h incubation period, each well was manually examined to determine the number of cell divisions: one cell indicated no division, and more cells indicated that the cell had divided.

#### Mouse serial CFU assays

A total of 300 mouse HSCs (Lineage^neg^, cKit^pos^, Sca-1^pos^, CD150^pos^, CD48^neg^, CD34^neg^) were sorted into 1 ml of MethoCult M3434 (StemCell Technologies) and plated for subsequent culture. Seven days after plating, the number of colonies was counted (colonies were defined as consisting of >300 cells). For second and third platings, 10^4^ cells were replated in MethoCult M3434 (StemCell Technologies). Colony formation resulting from these subsequent platings was assessed approximately 3 and 5 days after the second and third rounds, respectively.

#### HSC transplantation experiments

A total of 500 HSCs (Lineage^neg^, cKit^pos^, Sca-1^pos^, CD150^pos^, CD48^neg^, CD34^neg^) were obtained from CD45.2 mice that had experienced MI surgery, along with their respective control group (sham surgery). These cells were transplanted into fully irradiated CD45.1 (Ly5.1) mice (cumulative dose of 4.5 Gy + 5 Gy), together with 4 × 10^5^ supportive spleen cells from age-matched CD45.1/2 mice. Transplantation was performed by tail vein injection within 24 h after irradiation. CD45.2-donor cell contribution was monitored in PB samples collected from the submandibular vein 4, 8, 12 and 16 weeks after transplantation. CD45.2 chimerism was assessed using flow cytometry with the following antibodies (all obtained from BioLegend): CD45.1-FITC (cat. no. 110706, 1:500), CD45.2-PB (cat. no. 109820, 1:500), CD11b-APC/Cy7 (cat. no. 101226, 1:1,000), Gr1-APC (cat. no. 108412, 1:1,000), CD8a-PE/Cy5 (cat. no. 100709, 1:1,000), CD4-PE/Cy5 (cat. no. 100513, 1:1,000) and B220-AF700 (cat. no. 103232, 1:500). After 16 weeks, the mice were euthanized and their BM (hip bone, tibia and femur) was subjected to analysis.

### Characterization of human HSPCs

#### Samples from patients with MI

Sternal BM was collected during surgical procedures. Exclusion criteria comprised patients with active cancer or haematological disorders, individuals who had received chemotherapy or radiation therapy, and those with acute infections. The inclusion criteria included patients scheduled for CABG surgery who had a preserved left ventricular EF greater than 45% and showed no signs of heart failure, for example proBNP >1,000 pg ml^−1^. The filtered patients were categorized into two groups: (1) control patients, with no history of MI, and (2) patients with MI, with a documented history of MI. Clinical characteristics such as age, gender, left ventricular EF, smoking status and proBNP were closely matched between the two groups (Supplementary Table 1).

#### Ethical considerations

This study was conducted in accordance with the ethical standards and guidelines established by the Ethical Review Board of Freiburg. Ethical approval for the study protocol (ethics approval number 388/19) was obtained from the Ethical Review Board of Freiburg on 16 January 2020. All experimental procedures involving human patients were performed in compliance with the relevant laws and institutional guidelines. Informed consent was obtained from all human participants involved in the study.

#### Human single-cell division assays on in vitro treated cells

Individual HSPCs (Lineage^neg^, CD38^neg^, CD34^pos^) or HSCs (Lineage^neg^, CD38^neg^, CD34^pos^, CD45RA^neg^) were sorted into 72-well Terasaki plates (Greiner Bio-One) containing StemSpan SFEM II medium (StemCell Technologies) supplemented with StemSpan CD34^pos^ Expansion Supplement (StemCell Technologies). For in vitro cell cultivation, at-RA (2.5 μM final concentration), 4-oxo-RA (2.5 μM final concentration) or the respective volume of DMSO was added to the culture medium. After a 48-h incubation period, each well was manually examined to determine the number of cell divisions: one cell indicated no division, and more cells indicated that the cell had divided.

#### Human serial CFU assays

A total of 1,000 HSPCs (Lineage^neg^, CD38^neg^, CD34^pos^) were sorted into 1 ml of MethoCult H4435 (StemCell Technologies) and plated for subsequent culture. Seven days after plating, the number of colonies was counted (colonies were defined as consisting of >300 cells). A total of 1,000 cells were replated in MethoCult H4435 (StemCell Technologies) and quantified after 7 days.

#### Human CFU assays on in vitro treated human HSPCs

A total of 1,000 HSPCs (Lineage^neg^, CD38^neg^, CD34^pos^) or HSCs (Lineage^neg^, CD38^neg^, CD34^pos^, CD45RA^neg^) were sorted into 96-well low-attachment plates containing StemSpan SFEM II medium (StemCell Technologies) supplemented with StemSpan CD34^pos^ Expansion Supplement (StemCell Technologies). Cells were stained using the following antibodies (all obtained from BioLegend): CD34-FITC (cat. no. 343603, 1:200) or CD34-APC/Cy (cat. no. 343513, 1:100), CD38-PE/Cy7 (cat. no. 356607, 1:200), Lineage-APC (CD3, CD14, CD16, CD19, CD20, CD56; cat. no. 348803, 1:200) or CD45RA-BV421 (cat. no. 304129, 1:100).

For in vitro treatments, cells were cultured for a period of 72 h using a concentration of at-RA (2.5 μM final), 4-oxo-RA (2.5 μM final) or the corresponding volume of DMSO. Subsequently, cells were transferred into 1 ml of MethoCult H4435 (StemCell Technologies) and serial CFU assay was performed as described above.

#### In vitro treatments of human monocytes

Human PB mononuclear cells were processed using the EasySep Human Monocyte Enrichment Kit (StemCell Technologies) to isolate monocytes for culture. Cells were cultured in RPMI 1640 medium supplemented with 1× l-glutamine, 10% FBS and 1× penicillin–streptomycin. For in vitro treatments, human monocytes were cultured in a medium containing a final concentration of 0.2 μM at-RA or 4-oxo-RA. After 16 h of culture, culture supernatant was collected for the cytokine secretion assay (human inflammatory cytokine panel obtained from BioLegend). For flow cytometry analysis and bulk RNA-seq analysis, cells were cultured for 24 h.

#### CellROX staining

Cells were incubated at 37 °C with CellROX Deep Red (1:500, Invitrogen) in their respective media for 30 min, according to the manufacturer’s instructions. Cells were washed three times with PBS and subsequently stained for FACS analysis on the BD LSRFortessa Cell Analyzer (BD Biosciences).

#### NBSGW transplantations

In total, 10,000 BM HSPCs (Lineageneg, CD34^pos^) were sorted and injected via tail vein into 6- to 12-week-old female NBSGW mice. Recipient mice were irradiated with 1 Gy and injected within 24 h after irradiation. CD45.2-donor cell contribution was monitored in PB collected from the submandibular vein at 6, 12, 18, 24 and 36 weeks after transplantation. Human CD45.2 chimerism was assessed using flow cytometry with the following antibodies: CD45-FITC (human; BioLegend, cat. no. 304005, 1:200) and CD45-PE (mouse; BioLegend, cat. no. 103105, 1:500). After 24 weeks, the mice were euthanized, and their BM (hip bone, tibia and femur) was subjected to analysis. A mouse was classified as engrafted if the human CD45-positive percentage was greater than or equal to 0.3%.

### Bulk RNA-seq

#### Nucleic acid extraction protocol

Cells were sorted into RNA lysis buffer (Arcturus PicoPure RNA Isolation Kit, Applied Biosystems) and then stored at −80 °C until further use. RNA isolation was conducted using the Arcturus PicoPure RNA Isolation Kit (Applied Biosystems) following the manufacturer’s guidelines. DNase treatment was carried out using the RNase-Free DNase Set (Qiagen). The resulting total RNA was utilized for generating cDNA libraries.

#### Nucleic acid library construction protocol

cDNA libraries were generated using SMARTseq v4 (Takara Bio). Amplification cycles were adjusted accordingly to RNA input amount. For HSCs, 13 cycles of amplification were performed. For cardiac cell populations, 12–14 cycles of amplification were performed. To produce uniquely and dually barcoded sequencing libraries from the cDNA libraries, the NEBNext Ultra II FS DNA library kit was utilized. This involved fragmenting 5 ng of the cDNA library for 22.5 min, followed by adaptor ligation and library amplification using cycle numbers determined by the amount of input material.

#### Nucleic acid sequencing

Libraries underwent sequencing on the Illumina NovaSeq platform, generating 45–55 million reads depth with 100-bp paired-end sequencing.

#### Population RNA-seq analysis method, low-level processing

Raw FASTQ files underwent alignment against the mm10 or hg38 reference genome using the mRNA-seq tool of the bioinformatics pipeline snakePipes v.2.5.2 (ref. ^65^). Within this tool, the Alignment mode was utilized for mapping the sequenced reads via STAR (STAR_2.7.4a)^66^, and expression counts were quantified using featureCounts^67^. Data quality was evaluated by Deeptools QC v3.3.2 (ref. ^68^). Genes with an average expression exceeding 100 counts in at least one condition were specifically selected for further analysis. To assess differential expression, DESeq2 was used^69^ and results were considered statistically significant at a false discovery rate of 0.1.

#### Population RNA-seq analysis method, downstream analysis

The expression of previously published gene signatures was assessed by GSEA by using the fgseaMultilevel function from the fgsea package with default parameters^70^, filtering significant pathways at a false discovery rate equal to 0.1. Specific signature enrichment profiles were generated with the gseaplot2 function^71^. The resulting normalized enrichment score (NES) and *P*-adjusted value of selected signatures were plotted using ggplot2 (ref. ^72^). In mouse-derived datasets, the enrichment of reactome pathways^73^, HSC/MPP^60^, activated/dormant HSC^19^, MolO/NoMo HSC^74^, RA metabolism^20^, inflammatory/reparatory-macrophage^75^, angiogenesis hallmark, wound healing (GO:0042060), extracellular matrix (GO:0031012) and inflammatory response (GO:0006954) signatures in pairwise comparisons was assessed. In the analysis of human data, LT(long-term)-HSC/ST(short-term)-HSC^76^, quiescent/activated LT-HSC^77^, HSC differentiation (GO:1902036), cell cycle (hsa04110), IFN-γ response (M5913) and inflammatory/reparatory-macrophage^78^ signatures were evaluated. In the dataset of cardiac differentiated cell populations, the average variance stabilising transformed (VST) expression values of selected genes were represented using the pheatmap package^79^. For the RA signalling translatability analysis, DEGs from our previously published mouse 4-oxo-RA and at-RA HSC treatments (adjusted *P* value <0.1)^20^ were translated to human gene symbols and depicted in volcano plots of our human data, when commonly up- or downregulated. GSEA of mouse direct target genes of both RA metabolites^20^ was also performed in our human dataset.

The raw data were deposited in ArrayExpress and are available under the accession numbers E-MTAB-13505, E-MTAB-13506, E-MTAB-13508 and E-MTAB-14660.

### scRNA-seq

#### Nucleic acid library and sequencing

scRNA-seq experiments were performed on 25,000 mouse BM HSPCs (Lineage^neg^, Sca1^pos^, cKit^pos^) or cardiac myeloid cells, 7,000 to 10,000 human BM HSPCs (Lineage^neg^, CD38^neg^, CD34^pos^) or 3,000–20,000 human BM monocytes. Sorted cells in PBS supplemented with 2% BSA were pelleted at 300*g* for 5 min at 4 °C and washed once in ice-cold PBS containing 0.5% BSA. Cells were resuspended in PBS with 0.5% BSA to a maximum cell concentration of 390 cells μl^−1^ and placed on ice. Cell viability was assessed by trypan blue staining. A cell suspension aliquot (2 μl) was diluted 1:2 in 0.4% trypan blue solution, incubated for 2 min on a microscope slide and analysed under a microscope. All samples for scRNA-seq analysis showed more than 90% viable cells. scRNA-seq was performed on the 10x Genomics platform using the Chromium Next GEM Single Cell 3′ Reagent Kit v3.1 dual index (10x Genomics, PN-1000268) following the manufacturer’s instructions. A total of 43 μl of cell suspension was loaded onto a chip G according to the manufacturer’s instructions, aiming for a targeted cell recovery of up to 10,000 cells. PCR cycles for cDNA and final library generation were adjusted according to the target cell recoveries. The quality of the obtained cDNA and final libraries was assessed by capillary electrophoresis (Fragment Analyzer, HS NGS Fragment Kit, Agilent). Sequencing was performed on a NovaSeq9000 device (Illumina) with a read length of 28–10–10–90 bp (R1–i5–i7–R2), aiming for a minimum sequencing depth of 20,000 read pairs per cell.

#### scRNA-seq analysis method, low-level processing

Raw unique molecular identifier (UMI)-based data files underwent mapping against the mm10 or hg38 reference genome using the scRNA-seq tool of the bioinformatics pipeline snakePipes v.2.5.2 with the 10xV3 mode^65^. With this tool, the reads were (1) mapped, (2) UMI-deduplicated and (3) counted using STARsolo, creating binary alignment and map (BAM) files and a Seurat object containing the gene counts^66^. Deeptools QC was used to assess the quality of these data^68^.

After data preprocessing, scRNA-seq analysis was performed with the R package Seurat^80^. The Seurat object was imported, and different cell filtering criteria, including a minimum number of counts and expressed genes per cell, were applied depending on the dataset to avoid empty droplets. Conversely, low-quality and dying cells with a percentage of mitochondrial mRNA exceeding 20% in HSPC datasets and 5% in Cd11b^pos^ datasets were excluded. Doublets were removed using the doubletFinder_v3 function from the DoubletFinder package^81^, assuming a maximum doublet formation rate of 7.6% (pK ≤0.076).

#### scRNA-seq analysis method, downstream analysis

A log transformation and normalization of the data were implemented before integrating the different samples to eliminate potential batch effects. Integration was performed using the functions SelectIntegrationFeatures, FindIntegrationAnchors and IntegrateData^82^. Linear dimensional reduction of the integrated dataset was performed through principal component analysis. After applying the JackStraw and Elbow plot methodologies from Seurat, a clustering analysis was carried out, selecting the initial 20 principal components. The identification of cell clusters was accomplished using the FindClusters method, and the results were visualized using the Uniform Manifold Approximation and Projection (UMAP) technique^83^. Clusters with a high doublet scoring were identified and filtered out using the doubletCells function from the scran package^84^. In addition, potential contaminations in the HSPC datasets were evaluated with the enrichment of LS-K signatures^85^ and differentiated cell markers, using AddModuleScore and FindAllMarkers, respectively. To annotate the filtered good-quality clusters, enrichment of previously published human BM HSPC markers^86,87^, mouse BM HSPC signatures^88^, human BM monocyte markers^89–91^ and mouse heart monocyte markers^92,93^ was assessed. In the cardiac 4-oxo-RA monocyte dataset of the Fgd5CreERT2 mouse model, filtered good-quality cells were projected against the previously analysed vehicle data using the FindTransferAnchors and MapQuery functions from Seurat. Predicted cell annotations were then represented in the vehicle UMAP reduction as reference.

The relative percentage of cells in each condition per cluster was quantified and visualized in a barplot, calculating the significance of these frequencies using Fisher’s exact tests^94^. In brief, we calculated the percentage of cells per annotated cluster relative to each condition and performed statistics in a separate manner to assess if there was a statistically significant association between cluster and condition. For this aim, a contingency table per cluster was created, reporting the relative percentage of cells from each condition in the cluster of interest and the respective percentage of cells that belonged to all other clusters. A two-sided Fisher’s exact test was then applied to these contingency matrices, and resulting *P* values were adjusted by the Bonferroni method. Cell density per separate condition was depicted using the MASS package^95^. To conduct RNA velocity analysis, spliced and unspliced reads were counted, and the dynamical model of the Python package scVelo was applied, as depicted in ref. ^96^.

The AddModuleScore function was used to illustrate the previously mentioned signature enrichment score. Gene expression and signature enrichment were represented using FeaturePlot, DimPlot and dotplot functions. Lists of cluster markers generated by the FindMarkers function (min.pct = 0.25, logfc.threshold = 0) were utilized for GSEA, as previously explained. Volcano plots of the DEGs were represented with EnhancedVolcano^97^. GO enrichment analysis of DEGs was performed with the enrichGO or compareCluster functions from the clusterProfiler package^98^.

The raw data are deposited in ArrayExpress and are available under accession numbers E-MTAB-13509, E-MTAB-13510, E-MTAB-13511, E-MTAB-13579 and E-MTAB-13580.

### Statistics and reproducibility

No statistical methods were used to predetermine sample sizes, but our sample sizes are similar to those reported in previous publications^20,76^. No data were excluded from the analyses. Data distribution was assumed to be normal, but this was not formally tested. The experiments were not randomized. The investigators were not blinded to allocation during experiments and outcome assessment.

### Reporting summary

Further information on research design is available in the Nature Portfolio Reporting Summary linked to this article.

## Online content

Any methods, additional references, Nature Portfolio reporting summaries, source data, extended data, supplementary information, acknowledgements, peer review information; details of author contributions and competing interests; and statements of data and code availability are available at 10.1038/s41556-025-01639-4.

## Supplementary information

Reporting Summary

Supplementary Table 1 Patient demographic and clinical characteristics comparison, related to Fig. 1.

Supplementary Table 2 Cluster markers and DEGs from scRNA-seq on human BM HSPCs upon MI, related to Fig. 1.

Supplementary Table 3 Cluster markers and DEGs from scRNA-seq on human BM monocytes upon acute and chronic MI, related to Fig. 1.

Supplementary Table 4 Cluster markers and DEGs from scRNA-seq on mouse cardiac CD11b^pos^ cells upon MI, related to Fig. 2.

Supplementary Table 5 Cluster markers and DEGs from scRNA-seq on 4-oxo-RA treated BM HSPCs upon MI, related to Fig. 4.

Supplementary Table 6 Cluster markers and DEGs from scRNA-seq on 4-oxo-RA treated spleen HSPCs upon MI, related to Fig. 4.

Supplementary Table 7 DEGs from scRNA-seq on mouse cardiac CD11b^pos^ cells upon MI comparing 4-oxo-RA and vehicle conditions, related to Fig. 5.

Supplementary Table 8 Cluster markers from scRNA-seq on 4-oxo-RA treated BM HSPC cells isolated from Rarβ-KO mice upon MI, related to Fig. 5.

Supplementary Table 9 DEGs from population RNA-seq on human BM HSPC cells from healthy donors and patients with MI upon 4-oxo-RA and at-RA treatment, and common mouse-human RA DEGs, related to Fig. 6.

## Source data

Source Data Fig. 1 Source data.

Source Data Fig. 2 Source data.

Source Data Fig. 3 Source data.

Source Data Fig. 4 Source data.

Source Data Fig. 5 Source data.

Source Data Fig. 6 Source data.

Source Data Extended Data Fig. 1 Source data.

Source Data Extended Data Fig. 2 Source data.

Source Data Extended Data Fig. 4 Source data.

Source Data Extended Data Fig. 6 Source data.

Source Data Extended Data Fig. 7 Source data.

Source Data Extended Data Fig. 8 Source data.

Source Data Extended Data Fig. 9 Source data.

## Data Availability

The raw data were deposited in ArrayExpress (https://www.ebi.ac.uk/arrayexpress) and are available under the accession numbers E-MTAB-13509, E-MTAB-13510, E-MTAB-13511, E-MTAB-13505, E-MTAB-13506, E-MTAB-13508, E-MTAB-13579, E-MTAB-13580 and E-MTAB-14660. Further information and requests for data, materials, resources and reagents should be addressed to T.H. or N.C.-W. Source data are provided with this paper.
