# Supplementary material for: Modulation of bone marrow haematopoietic stem cell activity as a therapeutic strategy after myocardial infarction: a preclinical study
Source: Nat Cell Biol. 2025 Apr 2;27(4):591–604. doi: 10.1038/s41556-025-01639-4 (PMC11991920; doi:10.1038/s41556-025-01639-4)
Supplement: Supplementary file 1 — Reporting Summary [file 41556_2025_1639_MOESM1_ESM.pdf]

nature portfolio

Corresponding author(s): Timo Heidt, Nina Cabezas-Wallscheid

Last updated by author(s): Dec 4, 2024

Reporting Summary

Nature Portfolio wishes to improve the reproducibility of the work that we publish. This form provides structure for consistency and transparency in reporting. For further information on Nature Portfolio policies, see our Editorial Policies and the Editorial Policy Checklist.

Statistics

For all statistical analyses, confirm that the following items are present in the figure legend, table legend, main text, or Methods section.

n/a	Confirmed	
☐	☒	The exact sample size (*n*) for each experimental group/condition, given as a discrete number and unit of measurement
☐	☒	A statement on whether measurements were taken from distinct samples or whether the same sample was measured repeatedly
☐	☒	The statistical test(s) used AND whether they are one- or two-sided<br>*Only common tests should be described solely by name; describe more complex techniques in the Methods section.*
☐	☒	A description of all covariates tested
☐	☒	A description of any assumptions or corrections, such as tests of normality and adjustment for multiple comparisons
☐	☒	A full description of the statistical parameters including central tendency (e.g. means) or other basic estimates (e.g. regression coefficient) AND variation (e.g. standard deviation) or associated estimates of uncertainty (e.g. confidence intervals)
☐	☒	For null hypothesis testing, the test statistic (e.g. *F*, *t*, *r*) with confidence intervals, effect sizes, degrees of freedom and *P* value noted<br>*Give P values as exact values whenever suitable.*
☒	☐	For Bayesian analysis, information on the choice of priors and Markov chain Monte Carlo settings
☐	☒	For hierarchical and complex designs, identification of the appropriate level for tests and full reporting of outcomes
☐	☒	Estimates of effect sizes (e.g. Cohen's *d*, Pearson's *r*), indicating how they were calculated

Our web collection on statistics for biologists contains articles on many of the points above.

Software and code

Policy information about availability of computer code

Data collection

snakePipes v.2.5.2 bioinformatics pipeline
Seurat, DoubletFinder, scran, MASS, EnhancedVolcano, clusterProfiler, fgsea, ggplot2 and pheatmap R packages
scVelo python package

Data analysis

Population RNA-seq analysis method: low-level processing:
Raw FASTQ files underwent alignment against the mm10 or hg38 reference genome using the mRNA-seq tool of the bioinformatics pipeline snakePipes v.2.5.2. Genes with an average expression exceeding 100 counts in at least one condition were specifically selected for further analysis. To assess differential expression, DESeq2 was employed.

Population RNA-seq analysis method: downstream analysis:
The expression of previously published gene signatures was assessed by Gene Set Enrichment Analysis (GSEA) by using the fgsea package. Specific signature enrichment profiles were generated with the gseaplot2 function. The average VST-transformed expression values of selected genes were represented using the pheatmap package.

ScRNA-seq analysis method: low-level processing:
Raw UMI-based data files underwent mapping against the mm10 or hg38 reference genome using the scRNA-seq tool of the bioinformatics pipeline snakePipes v.2.5.2 with the 10xV3 mode. Following data preprocessing, scRNA-seq analysis was performed with the R package Seurat. The Seurat object was imported and different cell filtering criteria, including a minimum number of counts and expressed genes per cell to avoid empty droplets, as well as low-quality and dying cells with a high percentage of mitochondrial mRNA. Doublets were removed using the doubletFinder_v3 function from the DoubletFinder package.

ScRNA-seq analysis method: downstream analysis:
A log transformation and normalization of the data were implemented before integrating the different samples to eliminate potential batch effects. Integration was performed using the functions SelectIntegrationFeatures, FindIntegrationAnchors, and IntegrateData. Linear dimensional reduction of the integrated dataset was performed through Principal Component Analysis (PCA). The identification of cell clusters was accomplished using the FindClusters method, and the results were visualized using the Uniform Manifold Approximation and Projection (UMAP) technique. Clusters with a high doublet scoring were identified and filtered out using the scran package. Additionally, potential contaminations in the HSPC datasets were evaluated with the enrichment of LS-K signatures and differentiated cell markers. To annotate the filtered good quality clusters, enrichment of previously published markers and signatures was assessed. In the cardiac 4-oxo-RA monocyte dataset of the Fgd5CreERT2 mouse model, filtered good quality cells were projected against the previously analyzed Vehicle data using the FindTransferAnchors and MapQuery functions from Seurat.
The relative percentage of cells in each condition per cluster was visualized in a barplot, calculating significance with Fisher tests. Cell density was depicted using the MASS package. To conduct RNA velocity analysis, the python package scVelo was applied. The AddModuleScore function was employed to illustrate signature enrichment score. Gene expression and signature enrichment were represented using FeaturePlot, DimPlot, and dotplot functions. Lists of cluster markers generated by the FindMarkers function were utilized for GSEA. Volcano plots of the differentially expressed genes (DEGs) were represented with EnhancedVolcano. Gene ontology (GO) enrichment analysis of DEGs was performed with the clusterProfiler package.

For manuscripts utilizing custom algorithms or software that are central to the research but not yet described in published literature, software must be made available to editors and reviewers. We strongly encourage code deposition in a community repository (e.g. GitHub). See the Nature Portfolio guidelines for submitting code & software for further information.

Data

Policy information about availability of data

All manuscripts must include a data availability statement. This statement should provide the following information, where applicable:

- Accession codes, unique identifiers, or web links for publicly available datasets
- A description of any restrictions on data availability
- For clinical datasets or third party data, please ensure that the statement adheres to our policy

The raw data were deposited in ArrayExpress (https://www.ebi.ac.uk/arrayexpress) and are available under the accession numbers E-MTAB-13509, E-MTAB-13510, E-MTAB-13511, E-MTAB-13505, E-MTAB-13506, E-MTAB-13508, E-MTAB-13579, E-MTAB-13580, E-MTAB-14660.

Research involving human participants, their data, or biological material

Policy information about studies with human participants or human data. See also policy information about sex, gender (identity/presentation), and sexual orientation and race, ethnicity and racism.

Reporting on sex and gender	The study incorporates samples from both male and female sexes. There are no exclusion criteria based on sex. However, due to the nature of myocardial infarction primarily affecting men and sample availability, there is a bias towards male samples, with 1 female and 39 male human patient samples. Gender information has not been collected.
Reporting on race, ethnicity, or other socially relevant groupings	Race or ethnicity data has not been collected.
Population characteristics	Detailed population characteristics of the human research participants are collected in Supplementary Table 1.
Recruitment	We collected sternal bone marrow biopsies from >100 patients undergoing cardiac surgery. Of note, patients with a history of cancer, hematological diseases, prior chemotherapy or radiation therapy, infectious diseases and acute infections were not included in this cohort. We then selected patients with coronary artery bypass grafting (CABG) surgery and excluded cases with heart failure by means of reduced left ventricular ejection fraction (EF ≤ 45%) or other signs of cardiac congestion (e.g. proBNP > 1000 pg/ml). A total of 40 biopsies were used in the study with patients falling into the category of either (1) control samples from patients with chronic coronary artery disease (CAD) but no history of MI, and (2) samples from patients with a history of MI. To ensure comparability between the control and MI group, clinical patient characteristics such as age, gender, and relevant health metrics were matched.
Ethics oversight	This study was conducted in accordance with the ethical standards and guidelines established by the Ethical Review Board of Freiburg. Ethical approval for the study protocol (Ethics Approval Number: 388/19) was obtained from the Ethical Review Board of Freiburg on January 16, 2020.

Note that full information on the approval of the study protocol must also be provided in the manuscript.

Field-specific reporting

Please select the one below that is the best fit for your research. If you are not sure, read the appropriate sections before making your selection.

☒ Life sciences ☐ Behavioural & social sciences ☐ Ecological, evolutionary & environmental sciences

For a reference copy of the document with all sections, see nature.com/documents/nr-reporting-summary-flat.pdf

Life sciences study design

All studies must disclose on these points even when the disclosure is negative.

Sample size	Sample size was determined based on extensive experience with similar experiments in our laboratory (Cabezas-Wallcheid et a., 2014 and 2017; Sommerkamp et al., 2020 and 2021; Renders et al., 2021; Schönberger et al., 2022; Zhang et al., 2022).
Data exclusions	Data exclusion for human patients is detailed in the previous recruitment section.
Replication	Replication was successful in every attempt. Key and possible experiments were performed at least twice, exact number of independent experiments with several biological replicates are shown in figure legends.
Randomization	All patients samples/mice were analyzed and allocated randomly.
Blinding	No blinding experiments were needed, as values were quantitative comparisons as determined by software and measurement.

Behavioural & social sciences study design

All studies must disclose on these points even when the disclosure is negative.

Study description	*Briefly describe the study type including whether data are quantitative, qualitative, or mixed-methods (e.g. qualitative cross-sectional, quantitative experimental, mixed-methods case study).*
Research sample	*State the research sample (e.g. Harvard university undergraduates, villagers in rural India) and provide relevant demographic information (e.g. age, sex) and indicate whether the sample is representative. Provide a rationale for the study sample chosen. For studies involving existing datasets, please describe the dataset and source.*
Sampling strategy	*Describe the sampling procedure (e.g. random, snowball, stratified, convenience). Describe the statistical methods that were used to predetermine sample size OR if no sample-size calculation was performed, describe how sample sizes were chosen and provide a rationale for why these sample sizes are sufficient. For qualitative data, please indicate whether data saturation was considered, and what criteria were used to decide that no further sampling was needed.*
Data collection	*Provide details about the data collection procedure, including the instruments or devices used to record the data (e.g. pen and paper, computer, eye tracker, video or audio equipment) whether anyone was present besides the participant(s) and the researcher, and whether the researcher was blind to experimental condition and/or the study hypothesis during data collection.*
Timing	*Indicate the start and stop dates of data collection. If there is a gap between collection periods, state the dates for each sample cohort.*
Data exclusions	*If no data were excluded from the analyses, state so OR if data were excluded, provide the exact number of exclusions and the rationale behind them, indicating whether exclusion criteria were pre-established.*
Non-participation	*State how many participants dropped out/declined participation and the reason(s) given OR provide response rate OR state that no participants dropped out/declined participation.*
Randomization	*If participants were not allocated into experimental groups, state so OR describe how participants were allocated to groups, and if allocation was not random, describe how covariates were controlled.*

Ecological, evolutionary & environmental sciences study design

All studies must disclose on these points even when the disclosure is negative.

Study description	*Briefly describe the study. For quantitative data include treatment factors and interactions, design structure (e.g. factorial, nested, hierarchical), nature and number of experimental units and replicates.*
Research sample	*Describe the research sample (e.g. a group of tagged Passer domesticus, all Stenocereus thurberi within Organ Pipe Cactus National Monument), and provide a rationale for the sample choice. When relevant, describe the organism taxa, source, sex, age range and any manipulations. State what population the sample is meant to represent when applicable. For studies involving existing datasets, describe the data and its source.*
Sampling strategy	*Note the sampling procedure. Describe the statistical methods that were used to predetermine sample size OR if no sample-size calculation was performed, describe how sample sizes were chosen and provide a rationale for why these sample sizes are sufficient.*
Data collection	*Describe the data collection procedure, including who recorded the data and how.*
Timing and spatial scale	*Indicate the start and stop dates of data collection, noting the frequency and periodicity of sampling and providing a rationale for these choices. If there is a gap between collection periods, state the dates for each sample cohort. Specify the spatial scale from which the data are taken*

Data exclusions	*If no data were excluded from the analyses, state so OR if data were excluded, describe the exclusions and the rationale behind them, indicating whether exclusion criteria were pre-established.*
Reproducibility	*Describe the measures taken to verify the reproducibility of experimental findings. For each experiment, note whether any attempts to repeat the experiment failed OR state that all attempts to repeat the experiment were successful.*
Randomization	*Describe how samples/organisms/participants were allocated into groups. If allocation was not random, describe how covariates were controlled. If this is not relevant to your study, explain why.*
Blinding	*Describe the extent of blinding used during data acquisition and analysis. If blinding was not possible, describe why OR explain why blinding was not relevant to your study.*

Did the study involve field work? ☐ Yes ☐ No

Field work, collection and transport

Field conditions	*Describe the study conditions for field work, providing relevant parameters (e.g. temperature, rainfall).*
Location	*State the location of the sampling or experiment, providing relevant parameters (e.g. latitude and longitude, elevation, water depth).*
Access & import/export	*Describe the efforts you have made to access habitats and to collect and import/export your samples in a responsible manner and in compliance with local, national and international laws, noting any permits that were obtained (give the name of the issuing authority, the date of issue, and any identifying information).*
Disturbance	*Describe any disturbance caused by the study and how it was minimized.*

Reporting for specific materials, systems and methods

We require information from authors about some types of materials, experimental systems and methods used in many studies. Here, indicate whether each material, system or method listed is relevant to your study. If you are not sure if a list item applies to your research, read the appropriate section before selecting a response.

Materials & experimental systems

n/a	Involved in the study	
☐	☒	Antibodies
☒	☐	Eukaryotic cell lines
☒	☐	Palaeontology and archaeology
☐	☒	Animals and other organisms
☒	☐	Clinical data
☒	☐	Dual use research of concern
☒	☐	Plants

Methods

n/a	Involved in the study	
☒	☐	ChIP-seq
☐	☒	Flow cytometry
☒	☐	MRI-based neuroimaging

Antibodies

Antibodies used

Antibody Source Identifier
D8a-BV650 BioLegend 100742; RRID:AB_2563056
CD11b-BV650 BioLegend 101259; RRID:AB_2566568
Gr1-BV650 BioLegend 108442; RRID:AB_2686974
TER119-BV650 BioLegend 116235; RRID:AB_11204244
B220-BV650 BioLegend 103241; RRID:AB_11204069
CD4-BV650 BioLegend 563232; RRID:AB_2738083
CD8a-PE/Cy7 BioLegend 100722; RRID:AB_312761
CD11b-PE/Cy7 BioLegend 101216; RRID:AB_312799
Gr1-PE/Cy7 BioLegend 108416; RRID:AB_313381
TER119-PE/Cy7 BioLegend 116221; RRID:AB_2137789
B220-PE/Cy7 BioLegend 103222; RRID:AB_313005
CD4-PE/Cy7 BioLegend 100422; RRID:AB_2660860
cKit-BV711 BioLegend 105835; RRID:AB_2565956
cKit-BV421 BioLegend 105828; RRID:AB_11204256
cKit-PE BioLegend 105808; RRID:AB_313217
Sca1-APC/Cy7 BioLegend 108126; RRID:AB_10645327
CD150-PE/Cy5 BioLegend 115912; RRID:AB_493598
CD48-Pacific Blue BioLegend 103418; RRID:AB_756140
Ki67-PE Invitrogen 12-5698-80; RRID:AB_11149672

Gr1-APC BioLegend 108412; RRID:AB_313377
CD11b-APC/Cy7 BioLegend 101226; RRID:AB_830642
CD150-BV605 BioLegend 115927; RRID:AB_11204248
CD48-BV421 BioLegend 103428; RRID:AB_2650894
CD34-FITC BD Biosciences 553733; RRID:AB_395017
CD34-AF700 BD Biosciences 560518; RRID:AB_1727471
CD45.1-FITC BioLegend 110706; RRID:AB_313495
CD45.2-Pacific Blue BioLegend 109820; RRID:AB_492872
CD45.1-PE/Cy7 BioLegend 110730; RRID:AB_1134168
CD127-PE BioLegend 121112; RRID:AB_493509
CD16/32-APC BioLegend 101326; RRID:AB_1953273
CD8a-FITC BioLegend 100706; RRID:AB_312745
CD11b-FITC BioLegend 101206; RRID:AB_312789
Gr1-FITC BioLegend 108406; RRID:AB_313371
Ter119-FITC BioLegend 116206; RRID:AB_313707
B220-FITC BioLegend 103206; RRID:AB_312991
CD4-FITC BioLegend 100406; RRID:AB_312691
CD48-PE/Cy7 BioLegend 103424; RRID:AB_2075049
B220-AF700 BioLegend 103232; RRID:AB_493717
hLineage-APC (CD3, CD14, CD16, CD19, CD20, CD56) BioLegend 348803
hCD45-FITC BioLegend 304005; RRID: AB_314393
hCD34-FITC BioLegend 343504; RRID: AB_1731852
hCD38-PeCy7 BioLegend 303516; RRID: AB_1279235
Ly-6C-FITC BioLegend 128006; RRID: AB_1186135
Ly-6C-PE BioLegend 128008; RRID: AB_1186132
Ly-6C-BV650 BioLegend 128049; RRID: AB_2800630
CD45.2-BV650 BioLegend 109835; RRID: AB_11203374
CD11b-PE/Cy7 BioLegend 101226; RRID: AB_830642
Ly-6G-APC BioLegend 127614; RRID:AB_2227348
Ly-6G-PE BioLegend 127608; RRID: AB_1186099
Ly-6G-BV650 BioLegend 127641; RRID: AB_2565881
CD19-PE/Cy7 BioLegend 115520; RRID: AB_313655
CD19-PE BioLegend 115508; RRID: AB_313643
CD3-PerCP/Cy5.5 BioLegend 100218; RRID: AB_1595492
CD115-PE BioLegend 135506; RRID: AB_1937253
CD115-BV711 BioLegend 135515; RRID: AB_2562679
CD16/32-APC/Cy7 BioLegend 101328; RRID: AB_2104158

Validation

All flow cytometry were already established and commonly used in our laboratory and have been published multiple times by us and other groups, as described in Cabezas-Wallscheid et al., Cell 2017; Schoenberger et al., CSC 2022; Zhang et al., NCB 2022.

Eukaryotic cell lines

Policy information about cell lines and Sex and Gender in Research

Cell line source(s)

State the source of each cell line used and the sex of all primary cell lines and cells derived from human participants or vertebrate models.

Authentication

Describe the authentication procedures for each cell line used OR declare that none of the cell lines used were authenticated.

Mycoplasma contamination

Confirm that all cell lines tested negative for mycoplasma contamination OR describe the results of the testing for mycoplasma contamination OR declare that the cell lines were not tested for mycoplasma contamination.

Commonly misidentified lines
(See ICLAC register)

Name any commonly misidentified cell lines used in the study and provide a rationale for their use.

Palaeontology and Archaeology

Specimen provenance

Provide provenance information for specimens and describe permits that were obtained for the work (including the name of the issuing authority, the date of issue, and any identifying information). Permits should encompass collection and, where applicable, export.

Specimen deposition

Indicate where the specimens have been deposited to permit free access by other researchers.

Dating methods

If new dates are provided, describe how they were obtained (e.g. collection, storage, sample pretreatment and measurement), where they were obtained (i.e. lab name), the calibration program and the protocol for quality assurance OR state that no new dates are provided.

☐ Tick this box to confirm that the raw and calibrated dates are available in the paper or in Supplementary Information.

Ethics oversight

Identify the organization(s) that approved or provided guidance on the study protocol, OR state that no ethical approval or guidance was required and explain why not.

Note that full information on the approval of the study protocol must also be provided in the manuscript.

Animals and other research organisms

Policy information about studies involving animals; ARRIVE guidelines recommended for reporting animal research, and Sex and Gender in Research

Laboratory animals	NOD,B6.SCID Il2rγ-/-KitW41/W41 Jackson 26497<br>C57Bl/6J (CD45.2) MPI-IE<br>B6Ly5.1 (CD45.1) MPI-IE<br>B6Ly5.1 (CD45.1/2) MPI-IE<br>Rarb tm1Vgi/HsvJ (Rarb KO) Jackson 022999<br>C57BL/6N-Fgd5tm3(cre/ERT2)Djr/J (Fgd5-CreERT2) Jackson 027789<br>B6.Cg-Gt(ROSA)26Sortm14(CAG-tdTomato)Hze/J (Rosa26-LSL-tdTomato) Jackson 007914
Wild animals	No wild animals were used in this study.
Reporting on sex	Female mice between 8-12 weeks of age were used.
Field-collected samples	No field collected samples were used in this study.
Ethics oversight	Animal procedures followed approved protocols by German authorities and Regierungspräsidium Freiburg, under §4 (3) of the German Animal Protection Act, with animal protocol numbers 35-9185.81/G-19/32, 35-9185.81/G-19/112, 35-9185.81/G21/063 and 35-9185.81/G-22/102. Lineage tracing experiments were performed under protocol 23-048-PIL approved by the CEEA committee of Parc Científic de Barcelona.

Note that full information on the approval of the study protocol must also be provided in the manuscript.

Clinical data

Policy information about clinical studies

All manuscripts should comply with the ICMJE guidelines for publication of clinical research and a completed CONSORT checklist must be included with all submissions.

Clinical trial registration	*Provide the trial registration number from ClinicalTrials.gov or an equivalent agency.*
Study protocol	*Note where the full trial protocol can be accessed OR if not available, explain why.*
Data collection	*Describe the settings and locales of data collection, noting the time periods of recruitment and data collection.*
Outcomes	*Describe how you pre-defined primary and secondary outcome measures and how you assessed these measures.*

Dual use research of concern

Policy information about dual use research of concern

Hazards

Could the accidental, deliberate or reckless misuse of agents or technologies generated in the work, or the application of information presented in the manuscript, pose a threat to:

No	Yes	
☐	☐	Public health
☐	☐	National security
☐	☐	Crops and/or livestock
☐	☐	Ecosystems
☐	☐	Any other significant area

Experiments of concern

Does the work involve any of these experiments of concern:

No	Yes	
☐	☐	Demonstrate how to render a vaccine ineffective
☐	☐	Confer resistance to therapeutically useful antibiotics or antiviral agents
☐	☐	Enhance the virulence of a pathogen or render a nonpathogen virulent
☐	☐	Increase transmissibility of a pathogen
☐	☐	Alter the host range of a pathogen
☐	☐	Enable evasion of diagnostic/detection modalities
☐	☐	Enable the weaponization of a biological agent or toxin
☐	☐	Any other potentially harmful combination of experiments and agents

Plants

Seed stocks	*Report on the source of all seed stocks or other plant material used. If applicable, state the seed stock centre and catalogue number. If plant specimens were collected from the field, describe the collection location, date and sampling procedures.*
Novel plant genotypes	*Describe the methods by which all novel plant genotypes were produced. This includes those generated by transgenic approaches, gene editing, chemical/radiation-based mutagenesis and hybridization. For transgenic lines, describe the transformation method, the number of independent lines analyzed and the generation upon which experiments were performed. For gene-edited lines, describe the editor used, the endogenous sequence targeted for editing, the targeting guide RNA sequence (if applicable) and how the editor was applied.*
Authentication	*Describe any authentication procedures for each seed stock used or novel genotype generated. Describe any experiments used to assess the effect of a mutation and, where applicable, how potential secondary effects (e.g. second site T-DNA insertions, mosiacism, off-target gene editing) were examined.*

ChIP-seq

Data deposition

☐ Confirm that both raw and final processed data have been deposited in a public database such as GEO.

☐ Confirm that you have deposited or provided access to graph files (e.g. BED files) for the called peaks.

Data access links<br>*May remain private before publication.*	*For "Initial submission" or "Revised version" documents, provide reviewer access links. For your "Final submission" document, provide a link to the deposited data.*
Files in database submission	*Provide a list of all files available in the database submission.*
Genome browser session<br>(e.g. UCSC)	*Provide a link to an anonymized genome browser session for "Initial submission" and "Revised version" documents only, to enable peer review. Write "no longer applicable" for "Final submission" documents.*

Methodology

Replicates	*Describe the experimental replicates, specifying number, type and replicate agreement.*
Sequencing depth	*Describe the sequencing depth for each experiment, providing the total number of reads, uniquely mapped reads, length of reads and whether they were paired- or single-end.*
Antibodies	*Describe the antibodies used for the ChIP-seq experiments; as applicable, provide supplier name, catalog number, clone name, and lot number.*
Peak calling parameters	*Specify the command line program and parameters used for read mapping and peak calling, including the ChIP, control and index files used.*
Data quality	*Describe the methods used to ensure data quality in full detail, including how many peaks are at FDR 5% and above 5-fold enrichment.*
Software	*Describe the software used to collect and analyze the ChIP-seq data. For custom code that has been deposited into a community repository, provide accession details.*

Flow Cytometry

Plots

Confirm that:

☒ The axis labels state the marker and fluorochrome used (e.g. CD4-FITC).

☒ The axis scales are clearly visible. Include numbers along axes only for bottom left plot of group (a 'group' is an analysis of identical markers).

☒ All plots are contour plots with outliers or pseudocolor plots.

☒ A numerical value for number of cells or percentage (with statistics) is provided.

Methodology

Sample preparation

Flow cytometric heart analysis post MI
Following euthanasia of the mice, hearts were extracted to analyze the infarcted myocardium. Infarcted myocardial tissue was excised, minced, and subjected to enzymatic digestion using a mixture of collagenase I (450 U/ml), collagenase XI (125 U/ml), DNase I (26 U/ml), and hyaluronidase (60 U/ml) (all from Sigma-Aldrich). Enzymatic reaction was incubated at a temperature of 37°C and a rotation speed of 600 rpm for a duration of 1 hour and subsequently stopped by the addition of 30 ml of FACS buffer consisting of phosphate-buffered saline (PBS) containing 0.5% bovine serum albumin and 1% fetal bovine serum.
Cardiac cell suspension was stained using the following antibodies: Lineage-BV605 (CD19, CD90, CD4, CD8a, NK1.1, Ter119, CD49b), Ly6C-FITC, CD115-BV711, Ly6G-APC, CD11b-APC-Cy7, CD45.2-PB, and F4/80-Pe-Cy7.

Flow cytometric blood, spleen, and whole BM analysis post MI
Following the extraction of venous blood via tail vein puncture, mice were euthanized to collect spleen, femurs, tibiae, and pelvis for BM analysis.
Venous blood was collected using 5 mM EDTA (Sigma-Aldrich). The spleen was filtered through a 40 µm cell strainer to create a single-cell suspension. The BM was flushed and then filtered through a 40 µm cell strainer to create a single cell suspension. Cell suspensions were treated with 1x red blood cell lysis buffer (BioLegend) for subsequent staining.
Spleen, blood, and whole BM were stained using the following antibodies: Ly6C-FITC, CD115-BV711, B220-BV650, CD3-PerCP-Cy5.5, Ly6G-APC, CD11b-APC-Cy7 and CD45.2-PB. Additionally, in a separate staining procedure, spleen, blood, and BM were stained using the following antibodies: Lineage-BV650 (Gr1, CD11b, B220, Ter119, CD4, CD8a), cKit-BV711, Sca1-APC-Cy7, CD150-PeCy5, CD48-BV421, CD16/32-APC, and CD127-Pe-Cy7.

Enrichment of mouse HSPCs and isolation of HSCs
Murine BM cells were isolated from the femur, tibia, hip bone, and vertebrae by gentle crushing with mortar and pestle in PBS. Red blood cell lysis was performed with ACK Lysing Buffer (Thermo Fisher Scientific) for 5 minutes at room temperature (RT). Dynabeads Untouched Mouse CD4 Cells Kit (Invitrogen) was used for lineage negative enrichment according to the manufacturer's protocol. Briefly, the BM was stained with 1:4 dilution of the Lineage Cocktail for 30–60 minutes at 4 °C on a rotating wheel. Labelled cells were then incubated for 20 minutes with 400 µl of washed Dynabeads coated with polyclonal sheep anti-rat IgG per sample. Depletion of lineage cells was performed using a magnet. Lineage-depleted BM cells were stained with lineage markers (Gr1, CD11b, B220, Ter119, CD4, CD8a), ckit, Sca1, CD150, CD48 and CD34. Sorting was then performed using a FACS Aria II, III or FACSymphony (Becton Dickinson).
Subsequently, cells were collected into ice-cold PBS for reconstitution assays, Complete Stem Cell Medium (StemPro-34 SFM, Life Technologies, supplemented with 50 ng/ml SCF, 25 ng/ml TPO, 30 ng/ml Flt3-Ligand [all from Preprotech], 100 µg/ml Penicillin/Streptomycin, and 2 mM L-Glutamine [both from Gibco]) for experiments for in vitro culture, PBS with 2% BSA for scRNA-seq, RNA lysis buffer (Arcturus PicoPure RNA Isolation Kit (Applied Biosystems)) for population RNA-seq and stored at −80°C.

Cell cycle analysis
Following lineage depletion, erythrocyte-lysed BM was stained for HSC markers (Lineageneg, cKitpos, Sca-1pos, CD150pos, CD48neg, CD34neg). Subsequently, cells were fixed for 10 minutes at 4°C using BD Cytofix/Cytoperm Buffer (Becton Dickinson and Company) and intracellular Ki-67 (BD Biosciences) staining was performed using PermWash solution for at least 45 minutes at 4°C (Becton Dickinson and Company). Before proceeding with flow cytometry analysis, the cells were stained with DAPI (Sigma-Aldrich) at RT for a minimum of 20 minutes.

Instrument

For cell sorting: FACS Aria Fusion (Becton Dickinson)
For analysis: LSRII, LSR Fortessa (Becton Dickinson)

Software

Analysis was performed with FlowJo, statistical analysis with Graphpad Prism.

Cell population abundance

Population abundance is reported on Figures

Gating strategy

Examples for every gating strategy are provided both in methods and figures.

☒ Tick this box to confirm that a figure exemplifying the gating strategy is provided in the Supplementary Information.

Magnetic resonance imaging

Experimental design

Design type	*Indicate task or resting state; event-related or block design.*
Design specifications	*Specify the number of blocks, trials or experimental units per session and/or subject, and specify the length of each trial or block (if trials are blocked) and interval between trials.*
Behavioral performance measures	*State number and/or type of variables recorded (e.g. correct button press, response time) and what statistics were used to establish that the subjects were performing the task as expected (e.g. mean, range, and/or standard deviation across subjects).*

Acquisition

Imaging type(s)	*Specify: functional, structural, diffusion, perfusion.*
Field strength	*Specify in Tesla*
Sequence & imaging parameters	*Specify the pulse sequence type (gradient echo, spin echo, etc.), imaging type (EPI, spiral, etc.), field of view, matrix size, slice thickness, orientation and TE/TR/flip angle.*
Area of acquisition	*State whether a whole brain scan was used OR define the area of acquisition, describing how the region was determined.*

Diffusion MRI ☐ Used ☐ Not used

Preprocessing

Preprocessing software	*Provide detail on software version and revision number and on specific parameters (model/functions, brain extraction, segmentation, smoothing kernel size, etc.).*
Normalization	*If data were normalized/standardized, describe the approach(es): specify linear or non-linear and define image types used for transformation OR indicate that data were not normalized and explain rationale for lack of normalization.*
Normalization template	*Describe the template used for normalization/transformation, specifying subject space or group standardized space (e.g. original Talairach, MNI305, ICBM152) OR indicate that the data were not normalized.*
Noise and artifact removal	*Describe your procedure(s) for artifact and structured noise removal, specifying motion parameters, tissue signals and physiological signals (heart rate, respiration).*
Volume censoring	*Define your software and/or method and criteria for volume censoring, and state the extent of such censoring.*

Statistical modeling & inference

Model type and settings	*Specify type (mass univariate, multivariate, RSA, predictive, etc.) and describe essential details of the model at the first and second levels (e.g. fixed, random or mixed effects; drift or auto-correlation).*
Effect(s) tested	*Define precise effect in terms of the task or stimulus conditions instead of psychological concepts and indicate whether ANOVA or factorial designs were used.*

Specify type of analysis: ☐ Whole brain ☐ ROI-based ☐ Both

Statistic type for inference<br>(See Eklund et al. 2016)	*Specify voxel-wise or cluster-wise and report all relevant parameters for cluster-wise methods.*
Correction	*Describe the type of correction and how it is obtained for multiple comparisons (e.g. FWE, FDR, permutation or Monte Carlo).*

Models & analysis

n/a	Involved in the study
☐	☐ Functional and/or effective connectivity
☐	☐ Graph analysis
☐	☐ Multivariate modeling or predictive analysis

Functional and/or effective connectivity	*Report the measures of dependence used and the model details (e.g. Pearson correlation, partial correlation, mutual information).*
Graph analysis	*Report the dependent variable and connectivity measure, specifying weighted graph or binarized graph,*

Graph analysis	*subject- or group-level, and the global and/or node summaries used (e.g. clustering coefficient, efficiency, etc.).*
Multivariate modeling and predictive analysis	*Specify independent variables, features extraction and dimension reduction, model, training and evaluation metrics.*
